# Immunoassay–mass spectrometry to identify *Brucella melitensis*

**DOI:** 10.3389/fcimb.2025.1531018

**Published:** 2025-02-04

**Authors:** Amirreza Sharif, Ramin Bagheri Nejad, Alireza Ghassempour

**Affiliations:** ^1^ Medicinal Plants and Drugs Research Institute, Shahid Beheshti University, Tehran, Iran; ^2^ Razi Vaccine & Serum Research Institute, Agricultural Research, Education and Extension Organization, Karaj, Iran

**Keywords:** immunoassay-mass spectrometry, *Brucella* spp., magnetic nanoparticles, milk, MALDI-TOF MS

## Abstract

Two factors frequently impede accurate bacterial identification using matrix-assisted laser desorption ionization time-of-flight mass spectrometry (MALDI-TOF MS): inadequate bacterial abundance in real samples and bacterial combinations. For MALDI-TOF MS analysis and libraries for bacterial identification, time-consuming culture procedures are necessary to achieve sufficient concentration and isolation of a single bacterium. When dealing with hazardous bacteria like *Brucella*, which are more difficult to handle and cure, this problem becomes even more crucial. To overcome these obstacles, Fe_3_O_4_ magnetic nanoparticles (MNPs) linked with *Brucella*-specific antibodies and MALDI-TOF MS analysis have been used to create a quick and accurate technique for direct bacterial separation and identification in complex samples. This method allows MNPs to immune-selectively collect *Brucella* cells, which are then deactivated and ready for MALDI-TOF MS analysis by a formic acid/acetonitrile wash. Rabbits were used to manufacture brucella antibodies, which have effectively adsorbed onto the MNPs–protein A. Any particular *Brucella* bacteria found in the media might be absorbed by this MNPs–protein A–antibody immunoprobe. The concentration of *Brucella* bacterial cells increases the protein spectrum’s visibility by a factor of 10^3^, making it possible to quickly identify *Brucella* spp. without first growing them in cultural conditions. This method has been successfully used to achieve a limit of detection (LOD) of 50 CFU/mL in an aqueous medium and genuine sample—milk. The diagnostic time for this harmful bacterium is greatly decreased because the entire procedure from bacterial isolation to species identification is finished in less than 60 min. High sensitivity and specificity are demonstrated by the immunoassay–MS approach, as the spectral pattern it produces matches well-known databases like SPECLUST and Ribopeaks.

## Graphical Abstract

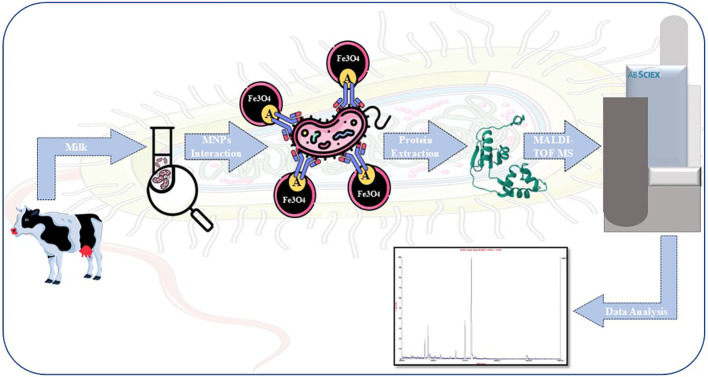

## 1 Introduction

The world’s health is being threatened by infectious germs, which have the capacity to start pandemics in the future (Nieto and Salvetti, 2014; Cloeckaert, 2024). Bacteria have historically caused a wide range of infectious diseases that impact people, animals, and plants (Dharmarajan et al., 2022; Tiedje et al., 2022; Glajzner et al., 2024). An estimated 420,000 deaths are attributed to foodborne pathogens alone each year, with the largest burden occurring in areas of poverty (Olivares et al., 2013; Schaumburg et al., 2019; de Oliveira et al., 2024). Therefore, bacterial illnesses must be properly diagnosed and treated in order to protect the public’s health (Zia and Alkheraije, 2023). Brucellosis is also known as “Mediterranean fever”, “undulant fever,” and “Malta fever”. Because of its low infectious dosage and ability to spread through inhaling contaminated aerosols, it is considered an infectious bacterial zoonosis (Qin et al., 2023; Zhang et al., 2024). Rapid and accurate identification of pathogenic microorganisms is necessary to comprehend these diseases (Wang et al., 2022; Kang et al., 2024). Matrix-assisted laser desorption ionization time-of-flight mass spectrometry (MALDI-TOF MS) utilizes a mass spectrum library derived from sufficient microbial proteins, allowing the matching of the obtained mass spectrum of one bacteria with known profiles for species identification (Cheng et al., 2016; Kurli et al., 2018; Rahi and Vaishampayan, 2020; Han et al., 2021; Lorente-Leal et al., 2022; Peng et al., 2022; Tsuchida and Nakayama, 2022; Becker and Lupetti, 2023; Foster and Khaiboullina, 2023).

Notable commercial kits include the developed fast BACpro^®^ II kit (Nittobo Medical Co., Tokyo, Japan) (Wang et al., 2016; Oviaño et al., 2021), the Vitek MS blood culture kit (bioMérieux, Inc.) (Nomura et al., 2020), and the Sepsityper^®^ kit (Bruker Daltonics) (Perše et al., 2022). For bacterial analysis in microbiology labs, including cultivation, inactivation, isolation, and data interpretation, the Food and Drug Administration (FDA) has authorized MALDI-TOF MS (Bauermeister et al., 2022; Li et al., 2022; Haider et al., 2023; Pastrone et al., 2023).

A potential method for quick and precise microbiological identification is the combination of MNPs and MALDI-TOF MS (Kumari et al., 2023). Detection limits as low as 10² CFU/mL are made possible by MNPs (Xiao et al., 2022; Abafogi et al., 2024). This technique improves operating speed and accuracy by streamlining processes by doing away with the requirement to culture samples, particularly for highly virulent pathogens such as *Brucella* (Ha and Kim, 2022). Furthermore, MNPs’ capacity to functionalize and attach selectively to bacterial cells enhances detection specificity in complicated biological samples, contributing to their high selectivity (Houser et al., 2024). Because of protein A’s high affinity for the Fc region of immunoglobulins (IgGs), antibodies on MNPs can be effectively immobilized, greatly enhancing their ability to ensnare target microorganisms (Xu et al., 2019).

Serological tests are usually preferred because determining the source of brucellosis is crucial due to its current threats and takes a lot of time, often requiring highly qualified specialists (Padilla et al., 2010). Serological techniques, however, frequently encounter obstacles like a lack of standardization, validation issues, and restrictions on precisely identifying the species and strain of *Brucella* implicated (Yagupsky et al., 2019).

In our last study, we used bioinformatics in conjunction with MALDI-TOF MS proteomics analysis to find biomarkers for brucellosis (Hamidi et al., 2022). Additionally, we used MALDI-TOF MS in conjunction with single-chain variable fragment (scFv) antibody-conjugated MNPs as highly sensitive and selective probes to quickly and precisely detect and identify the fig mosaic virus (Soleimani Mashhadi et al., 2020).

Using Fe₃O₄ MNPs modified with protein A and particular antibodies produced in rabbits, an immunoaffinity probe was created in this study to selectively isolate *Brucella* bacteria from contaminated samples at low concentration levels. This was followed by identification using the MALDI-TOF MS technique. This proposed technique increased the limit of detection (LOD) of *Brucella* bacteria by a factor of 1,000 in aqueous medium and milk samples.

## 2 Materials and methods

### 2.1 Material

#### 2.1.1 Chemicals and solutions

In this investigation, the following substances were used: CHEM LAB Co. (Zedelgem, Belgium) provided the sodium carbonate and sodium dodecyl sulfate (SDS). Sigma-Aldrich Co. (Altenburg, USA) provided the magnetic iron oxide nanoparticles (Fe_3_O₄ MNPs), acrylamide, N,N’-methylene bisacrylamide, sinapinic acid, and α-cyano-4-hydroxycinnamic acid (CHCA).

The following additional reagents were obtained from Merck Co. (Darmstadt, Germany): tetramethylethylenediamine (TEMED), Tris (hydroxymethyl) aminomethane, silver nitrate, tetraethyl orthosilicate (TEOS), N,N-dimethylformamide (DMF), succinic anhydride, disodium phosphate, potassium dihydrogen phosphate, glycine, ammonia solution (28%), acetonitrile (HPLC grade), absolute ethanol, formic acid, and trifluoroacetic acid (TFA).

The supplier of formaldehyde was Ghatranshimi Co. in Tehran, Iran. The supplier of ammonium persulfate (APS) was GE Healthcare Co. (Chicago, USA). We purchased 3-aminopropyltriethoxysilane (APTES), 1-ethyl-3-(3-dimethylaminopropyl) carbodiimide (EDC), and N-hydroxysuccinimide (NHS) from Exir GmbH Co. (Vienna, Austria). Lastly, BIOCHEM Chemopharma Co. (Burgundy, France) was the supplier of potassium chloride and sodium chloride.

### 2.2 Preparation of antibody immobilized on magnetic iron oxide nanoparticles

Initially, 100 mg of MNPs was subjected to a 10-min sonication. After adding ethanol (EtOH), water, ammonia, and TEOS, the mixture was sonicated for 3 h at room temperature. After that, ethanol was used to wash the MNPs. After adding APTES, water, ammonia, and EtOH, the mixture was sonicated for an hour at room temperature and then washed with ethanol to aminate the MNPs.

After the MNPs were cleaned, succinic anhydride was added, and the reaction was left to continue stirring all night. EDAC and NHS were added to the succinylated MNPs after they had been dissolved in 50 mM phosphate-buffered saline (PBS, pH 6.6) to activate the carboxyl groups (Mahmoud et al., 2005).

Following washing, the suspension was vortexed for 3 h and 2 mL of 500 ppm protein A solution was added (Ramandi et al., 2022). Two milliliters of 50 mM PBS (pH 6.6) was used to wash the MNPs three times for 10 min each time. For the SDS-PAGE analysis, 30 μL of each sample was kept. A Tris-HCl buffer (pH 8) was used to quench the MNPs’ unreacted sites.

**Figure 1:**
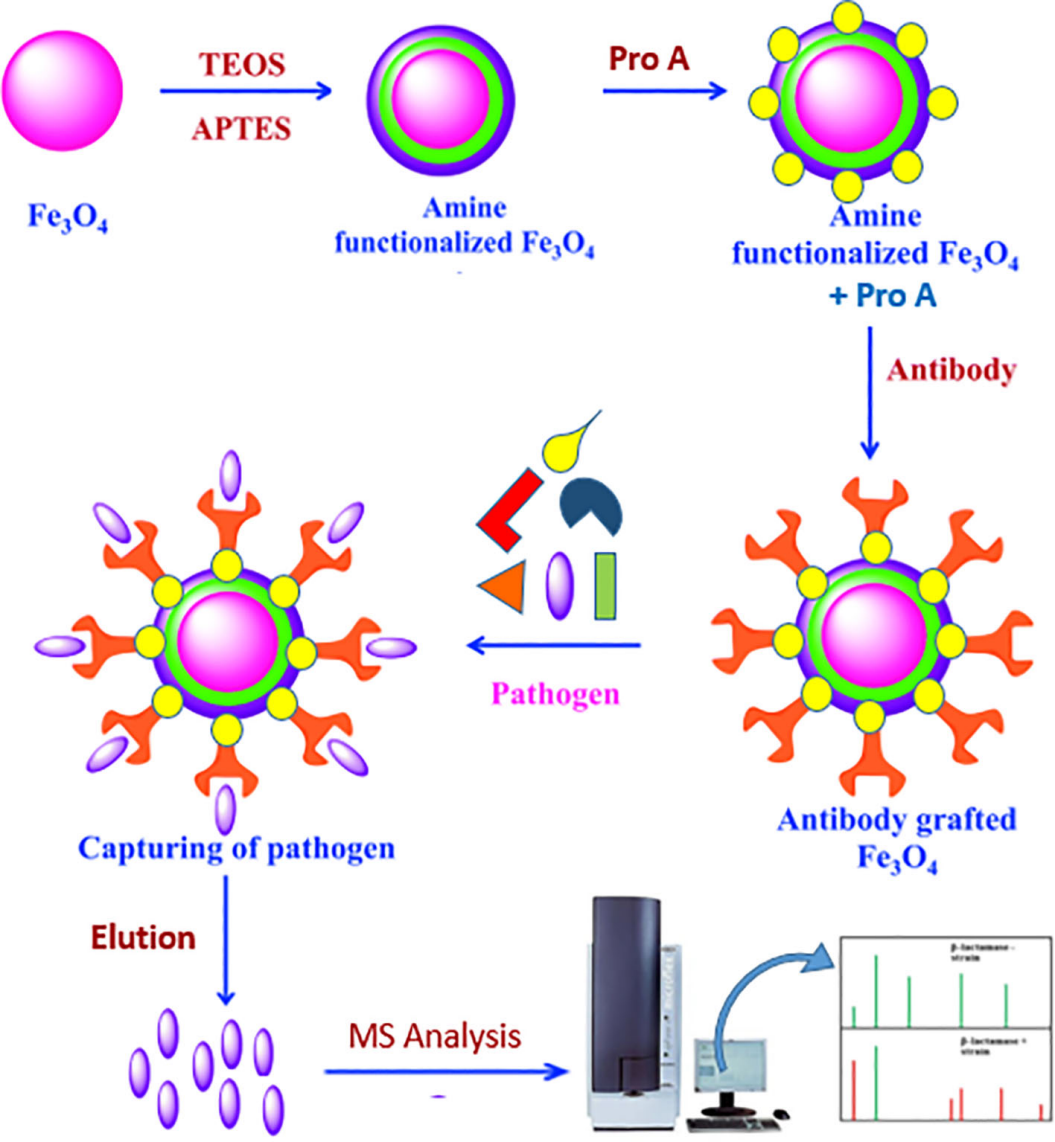
Activation of MNPs and immobilization of protein A and adsorption of antibody on it: TEOS (tetraethyl orthosilicate) and APTES (3-aminopropyltriethoxysilan).

The buffer was switched out for 50 mM PBS (pH 7.4) in order to encapsulate the antibody. After adding 2 mL of a 500 ppm *Brucella* antibody solution to the MNPs–protein A complex, the mixture was vortexed for 3 h. Two milliliters of 50 mM PBS (pH 7.4) was used to wash the MNPs three times for 10 min each time. Once more, 30 μL of every sample was saved for SDS-PAGE examination. For bacterial enrichment, the resultant MNPs–protein A–antibody complex was utilized (**Figure 1**).

### 2.3 SDS-PAGE analysis

Using the Bio-Rad system (Hercules, CA, USA), one-dimensional SDS-PAGE was performed. A 15% polyacrylamide gel made with Tris-glycine buffer was loaded with protein samples. For the best protein band separation, electrophoresis was carried out at a steady voltage.

To see the separated proteins, a silver nitrate staining procedure was used on the gel following electrophoresis. In short, the gel was sensitized in a sodium thiosulfate solution after being fixed in a methanol, acetic acid, and water solution to maintain the protein bands. After staining the gel with silver nitrate, it was developed with a developer solution based on formaldehyde until protein bands were visible. The gel was submerged in a stop solution to stop the reaction. High-sensitivity protein band detection was made possible by this procedure, guaranteeing that the separated proteins could be seen for further examination.

### 2.4 *Brucella* culture

The reference strain *Brucella melitensis* 16M was obtained from the Razi Vaccine and Serum Research Institute (Karaj, Iran). The bacteria were cultured on *Brucella* agar plates (BBL Microbiological Systems, Cockeysville, MD, USA), a selective medium specifically designed to support the growth of *Brucella* species. The plates were incubated under aerobic conditions at 37°C for 6 days to allow for sufficient bacterial growth.

To ensure optimal bacterial proliferation, the agar plates were prepared fresh and maintained in sterile conditions. Colony morphology was observed daily to confirm the growth characteristics of *B. melitensis* 16M, and any contamination was ruled out through visual inspection. After the incubation period, bacterial colonies were harvested under aseptic conditions and prepared for downstream applications, such as protein extraction, immunoassay development, or mass spectrometric analysis.

### 2.5 Produce antibody in rabbit

A fresh overnight culture was diluted in PBS to create a bacterial solution with a concentration of 10^8^ CFU/mL to generate antibodies against *B. melitensis*. Two doses of this suspension were administered to New Zealand White rabbits, with a 3-week gap between the first and booster shots. Two weeks following the booster injection, jugular vein blood samples were taken to extract antibody production. MNPs were employed to extract *B. melitensis* antibodies from the serum, which were then utilized for the following procedures.

### 2.6 Extraction of bacteria from samples with MNPs–protein A–antibody

The initial sample was diluted to reach a concentration of 50 billion bacteria after bacterial growth, then subsequent dilutions (50 to 5×10^8^ CFU/mL) were made. Different concentrations of MNPs–protein A–antibody were applied to 5×10^6^ CFU/mL of bacterial suspension to maximize bacterial separation. Bacteria attached to the MNPs–protein A–antibody conjugates were collected after 30 min of incubation at 37°C with constant shaking. They were then rinsed for 10 min with 1,000 μL of PBS (pH 7.4) and then again for 10 min with 1,000 μL of deionized water. After resuspending the particles in 15 μL of 70% formic acid and MS-grade acetonitrile, they were shaken to break down the bacterial cell walls and extract the proteins. The supernatant was spotted on a MALDI plate using the CHCA matrix for analysis.

### 2.7 MALDI-TOF MS analysis

An Applied Biosystems 4800 MALDI-TOF MS equipped with a Nd laser (200 Hz, AB Sciex, Canada) was used to obtain the mass spectra of bacterial proteins. CHCA (10.0 mg/mL in 2.5% TFA and 50% acetonitrile). The sample solution was added to the MALDI plate after 1 μL of the matrix solution. With a mass range of 2–20 kDa, the analysis was carried out in positive ion linear mode. Each sample received between 600 and 1,000 laser pulses, and the Data Explorer software (version 4.0) was used to create the average mass spectrum.

For bacterial inactivation and extraction, we employed a formic acid–ethanol extraction strategy. A bacterial suspension containing 6 to 10 colonies in 600 μL of water was vortexed for 10 s, followed by the addition of 1,000 μL of absolute ethanol. The mixture was carefully mixed and incubated at 20–25°C for 30 min and then centrifuged at 10,000 rpm for 10 min. The supernatant was removed, and the pellet was resuspended in 10 μL of 70% formic acid. Ten microliters of acetonitrile was added to ensure thorough mixing.

After centrifuging the mixture for 2 min at 11,000 rpm and 20–25°C, the supernatant was gathered and placed in a separate tube. The supernatant was put onto *Brucella* agar and cultured for 6 days at 37°C in order to evaluate the vitality of the bacteria. The effectiveness of the chemical procedures in deactivating *Brucella* spp. was validated by the lack of bacterial colonies.

## 3 Results

The SDS-PAGE analysis revealed successful immobilization of protein A on MNPs. The presence of protein A was confirmed in the supernatant, wash 1, and wash 2, indicating that protein A was effectively fixed on the MNPs and was ready for antibody binding to capture specific antigens (S1). Additionally, *Brucella* antibodies are produced in rabbits and purified via MNPs–protein A. The SDS-PAGE gel demonstrating the MNPs–protein A–antibody compared to pure antibodies is illustrated (S2). The non-covalent bond formed between protein A and the *Brucella* antibody allows for the potential purification of the *Brucella* antibody through elution from the MNPs–protein A–antibody surface.

A dilution of 5×10³ CFU/mL of *Brucella* bacteria was incubated with the antibody-coated substrate, and the results were compared to a substrate devoid of antibodies to verify the development of the antibody–antigen bond. The proteins of the *Brucella* bacteria that were isolated from the antibody-coated substrate produced the anticipated protein spectrum, while no such spectrum was seen from the substrate without antibodies. Additionally, MNPs–protein A–antibody was given to 5×10³ CFU/mL of *Escherichia coli* to verify its specificity, and no peaks were observed. Considering the necessity to optimize the amount of MNPs–protein A–antibody for isolation, we tested different substrate concentrations (0.5, 1, 2, and 5 mg/mL) using 5×10^3^ CFU/mL of *Brucella* bacteria (**Table 1**). Significant variations in protein spectrum intensity across the different substrate amounts led to the conclusion that 2 mg/mL was the ideal substrate concentration.

**Table 1:** Comparison amount of the ability of MNPs–protein A–antibody to achieve bacteria.

MNPs (mg/mL)	Intensity
0.5	64.3
1	264.0
2	987.5
5	1,085.8

The antibody-coated substrate was also kept in PBS buffer at 4°C for 3 months to evaluate its shelf life. Effective contact with bacteria was validated by post-storage analysis, which also showed that the produced MNPs–protein A–antibody remained functional for a minimum of 3 months.

To assess the pre-concentration effectiveness of the MNPs–protein A–antibody, both with the optimal quantity of MNPs–protein A–antibody after extraction using MALDI-TOF MS and without trapping the bacteria on the substrate, bacterial proteins were examined at various dilutions. The colony count in the initial suspension was 5×10^8^ CFU/mL. To extract the *Brucella* bacteria, several aqueous bacterial suspensions were made at dilutions ranging from 5×10⁸ to 50 CFU/mL. A LOD of 5×10^4^ and 50 CFU/mL in the aqueous environment without and with pre-concentration, respectively, was found in the mass spectra derived from these investigations.

To verify the analysis of *B. melitensis* 16M, the obtained mass spectra were compared to reference spectra, which showed a sizable number of similar peaks. They were generated in an aqueous medium to continue a series of dilutions that interacted with 2 mg/mL of the substrate, ranging from 5×10⁸ to 50 CFU/mL of the original bacterial suspension. After the proteins from the *Brucella* bacteria were extracted, the data were examined and are shown in **Table 2**. The average standard deviation (SD) was 12.3, and the linear discriminating range (LDR) was 10^7^, signifying a noteworthy 10^3^-fold increase in the concentration coefficient.

**Table 2:** Evaluate the pre-concentration efficacy of the MNPs–protein A–antibody, without trapping the bacteria on the substrate and with the optimized amount of MNPs–protein A–antibody following extraction via MALDI-TOF MS.

Bacterial dilution (CFU/mL)	Intensity Non-interaction ± *SD*	Intensity Interaction ± *SD*
5×10^8^	2923.4 ± 12.2	3536.1 ± 12.8
5×10^7^	1978.7 ± 11.4	2488.5 ± 13.7
5×10^6^	1696.0 ± 13.1	2430.4 ± 11.8
5×10^5^	1589.2 ± 12.6	1886.6 ± 12.3
5×10^4^	1071.1 ± 11.9	1785.7 ± 12.6
5×10^3^	–	1553.0 ± 11.2
5×10^2^	–	1433.9 ± 10.9
50	–	537.0 ± 13.4

**Figure 2:**
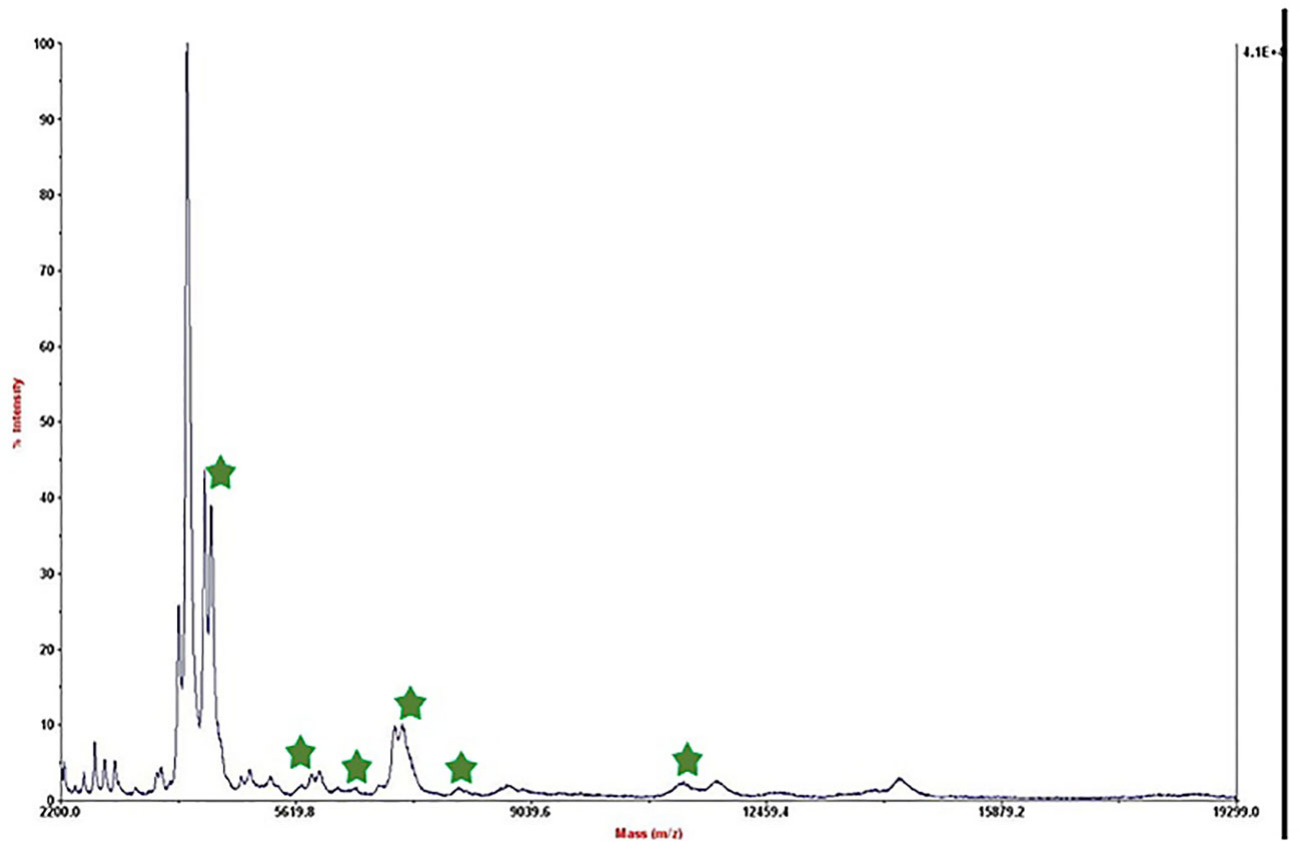
Mass spectrum of *B*. *melitensis* in 50 CFU/mL dilution in milk after interaction with MNPs–protein A–antibody.

We referred to the average total viable count in urban samples, which is reported to be between 10^8^ and 10^5^ CFU/mL (Berhe et al., 2020; Mukhopadhyay et al., 2024), in order to further modify this method for biological applications. Therefore, we made bacterial dilutions ranging from 5×10^4^ to 50 CFU/mL and added them to low-fat pasteurized milk as an actual sample analysis. Following the established procedure, we extracted the protein after permitting contact with the substrate. **Figure 2** displays the outcome of the 50 CFU/mL dilution analysis.

**Figure 3:**
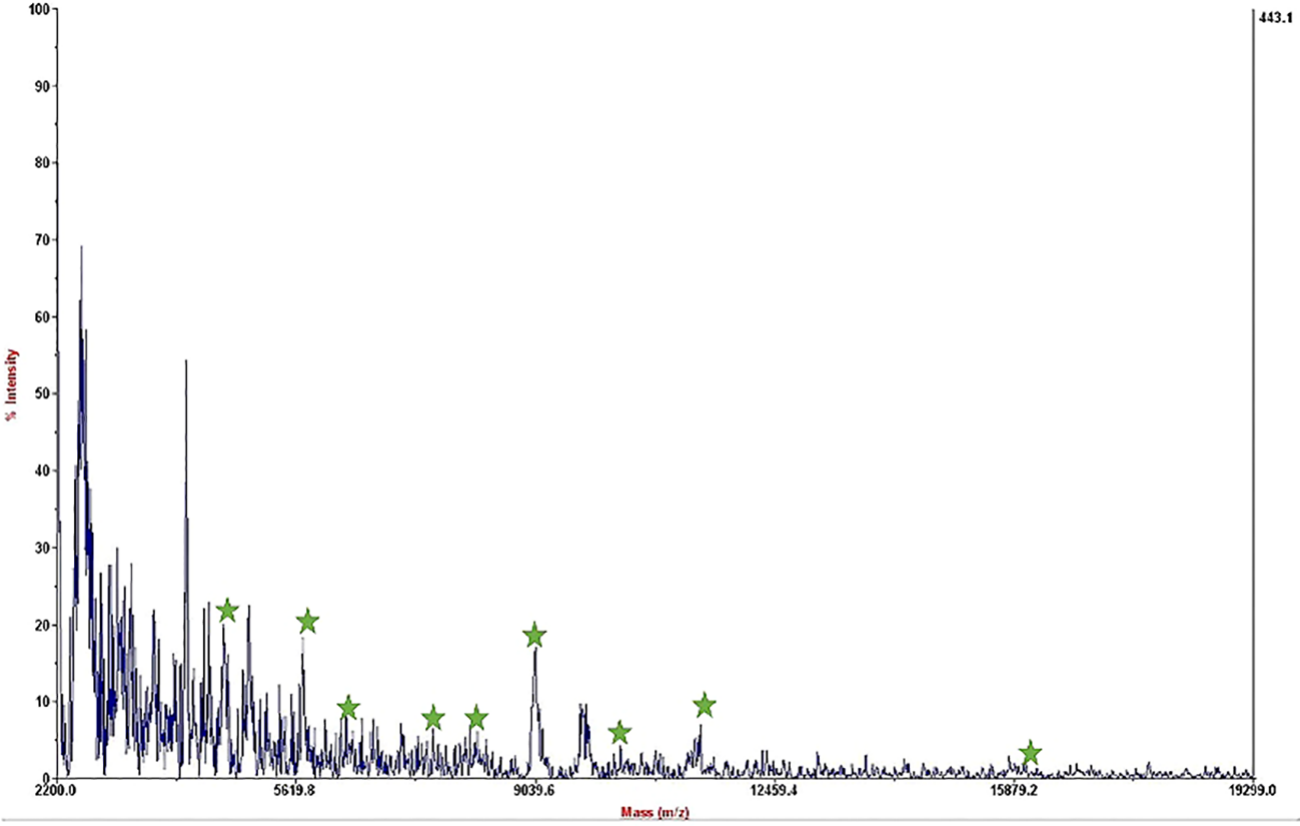
Mass spectrum of *B*. *melitensis* in 50 CFU/mL dilution in milk after interaction with MNPs–protein A–antibody and improvement.

The mass spectrum displayed significant interference from milk proteins (Šebela, 2022; Cuccato et al., 2022), prompting the exploration of several strategies to enhance the detection of bacterial protein peaks. The approaches employed included centrifugation of contaminated milk, followed by dilution of the sediment in water prior to interaction with the substrate (Punyapornwithaya et al., 2009; Tiedje et al., 2022), washing with a Tween-20-containing buffer, and adding additional washing steps (Barreiro et al., 2012). These methods were applied to bacterial dilutions of 5×10^4^ to 50 CFU/mL, resulting in notable improvements, as shown in **Figure 3**. Ongoing efforts are focused on optimizing the analysis of smaller bacterial quantities. It means that the LOD of this technique is 50 CFU/mL and we can detect bacteria in milk samples. The significance of this method lies in its potential for rapid and accurate pathogen identification, thereby aiding medical professionals and contributing to public health. This report details our progress to date, with continued work aimed at validating the method for practical applications in clinical settings.

## 4 Discussion

We found that 2 mg/mL of MNPs–protein A–antibody is the ideal concentration for efficiently isolating pathogens in complex matrices like milk, where proteins and lipids can make isolation difficult. Our findings concur with those of Xiao et al (Xiao et al., 2022), who emphasized the significance of optimal MNP concentrations for raising the sensitivity of detection. Using MNPs in conjunction with immunoassay methods, we were able to detect *B. melitensis* in milk and water samples with an LOD of 50 CFU/mL. The application of polydopamine-coated MNPs for automated sepsis detection was recently demonstrated by Zhang et al., who achieved remarkable sensitivity with detection limits as low as 10² CFU/mL across a variety of bacterial species in blood samples (Houser et al., 2024).

Similar to our strategy of using MNPs for bacterial collection from milk, this degree of sensitivity highlights how well MNPs work to capture and concentrate germs from complicated matrices. Furthermore, Chen et al. reported that MNPs grafted with antibodies can greatly increase detection sensitivity (Chen et al., 2022). Our findings that antibody-functionalized MNPs improve the overall sensitivity of bacterial protein spectra by a factor of 10³ detection and expedite the isolation procedure are corroborated by this evidence. When compared to traditional culture methods, our method of using MALDI-TOF MS in conjunction with MNPs provides rapid identification capabilities in less than 60 min, which is a significant reduction in the time needed for bacterial identification. For prompt clinical decision-making and efficient patient care, this quick turnaround is essential.

Additionally, the significant problem of low bacterial abundance in samples is resolved by integrating MNPs with MALDI-TOF MS. Furthermore, new research highlights the adaptability and efficiency of iron oxide-based MNPs in the identification and management of bacteria (Svadlakova et al (Svadlakova et al., 2020)). Improvements in magnetic nanoparticle-based microfluidic systems that allow for the quick and accurate identification of harmful bacteria in a variety of sample types, including food and water, were covered by Han et al (Han et al., 2021). Furthermore, Ha et al. described methods that increase sensitivity and specificity even in complex food matrices (Ha and Kim, 2022) and demonstrated how magnetic nanoparticles improve immunoassays for pathogen detection.

By offering prompt and precise bacterial identification that is essential for efficient patient care and treatment, this quick turnaround has the potential to completely transform clinical microbiology. Although this approach may have a significant impact on clinical microbiology, its wider application will require additional verification of its precision and consistency across a range of bacterial strains and clinical settings.

Adapting this technique to a range of diseases requires high-affinity antibodies that target specific pathogen indicators, such as lipopolysaccharides or cell wall proteins for bacteria and capsid or envelope proteins for viruses. These antibodies ensure the sensitivity and specificity of the immunoaffinity enrichment. The high-throughput, rapid, and cost-effective technique of MALDI-TOF mass spectrometry enhances bacterial identification. However, in settings with limited resources, its high initial costs, maintenance needs, and reliance on skilled personnel may make it less accessible. Continuous sample preparation and efficient processes are necessary for scalability. Despite these challenges, MALDI-TOF offers scalable, cost-effective diagnostics with exceptional mass accuracy and precision.

Although MNPs have several drawbacks when used in therapeutic settings, they provide intriguing applications for bacterial enrichment. It is difficult to achieve specificity across a variety of bacterial populations; thus, current research attempts to improve the identification of mixed pathogens by developing particular substrates based on patient clinical circumstances and protein biomarkers (Sandrin and Demirev, 2018; Yang et al., 2018; Han et al., 2021). The efficiency of magnetic separation is affected by factors such as flux and magnetic field strength, which can be improved by synthesizing smaller MNPs and their quantity optimization for bacterial extraction (Socas-Rodríguez et al., 2020). Furthermore, complex biological matrices often interfere with binding and enrichment processes, requiring preconcentration methods such as centrifugation to clean up samples (Xu et al., 2019). Moreover, implementing MNP-based methods in clinical settings requires compliance with regulatory standards, which presents challenges in standardizing protocols across laboratories (Takallu et al., 2024). It will be essential to highlight these challenges to advance the use of MNPs in routine microbiological diagnostics.

Using this method within the context of an automated diagnostic platform or a field-deployable kit, MS is thus a viable strategy for advancing microbial diagnostics. Future research should involve the development of automated magnetic separation techniques that will improve the method’s efficiency as shown by various studies that have used vancomycin- and allantoin-conjugated MNPs for the rapid concentration of bacteria from the complex samples (Han et al., 2021; Abafogi et al., 2024). In addition, the development of portable diagnostic kits that contain MNPs will help in the identification of pathogens in the affected regions (Wen et al., 2017). The specificity, sensitivity, and selectivity of MNPs can be increased further to capture a wider range of bacterial strains through the use of new conjugation techniques (Gao et al., 2016). In general, these developments can be viewed as having the capacity to enhance microbial diagnostic capabilities in the clinical setting with speed and efficiency.

## Data Availability

The datasets presented in this study can be found in online repositories. The names of the repository/repositories and accession number(s) can be found in the article/**Supplementary Material**.
