# Supplementary material for: Immunoassay–mass spectrometry to identify Brucella melitensis
Source: Front Cell Infect Microbiol. 2025 Feb 4;15:1531018. doi: 10.3389/fcimb.2025.1531018 (PMC11832529; doi:10.3389/fcimb.2025.1531018)
Supplement: Supplementary file 1 [file Table1.docx]

Supplementary:


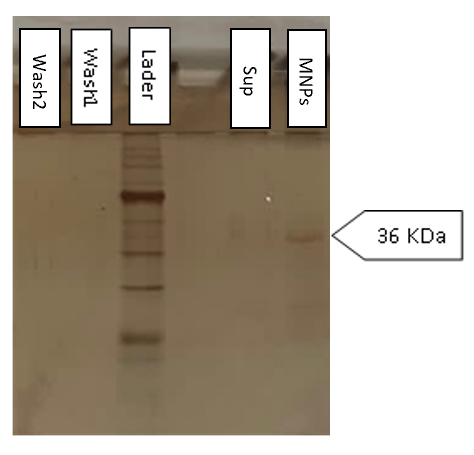


**Figure S1**: 15% SDS page gel prepared from MNPs containing pro A,

supernatant, washing 1 and washing 2


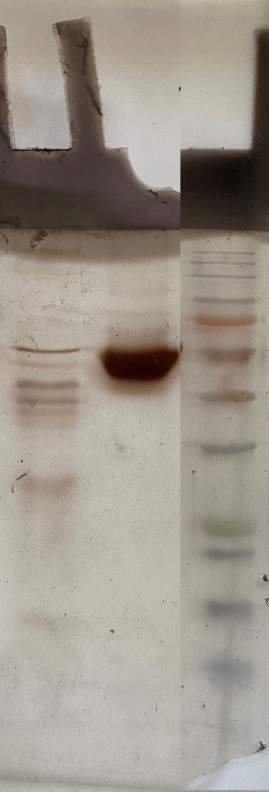

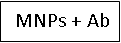

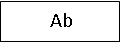

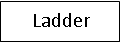


53 KDa

**Figure S2**: 15% SDS page gel prepared from MNPs containing Ab and pure Ab.


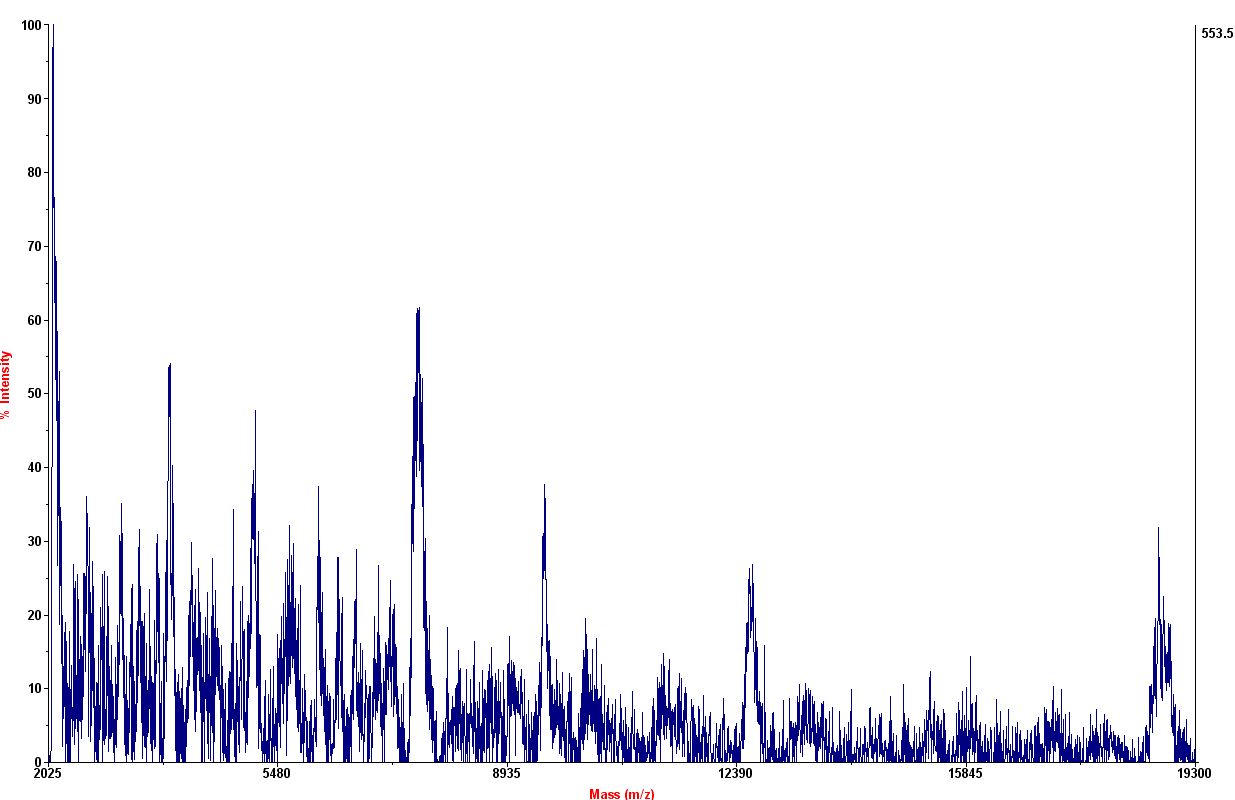

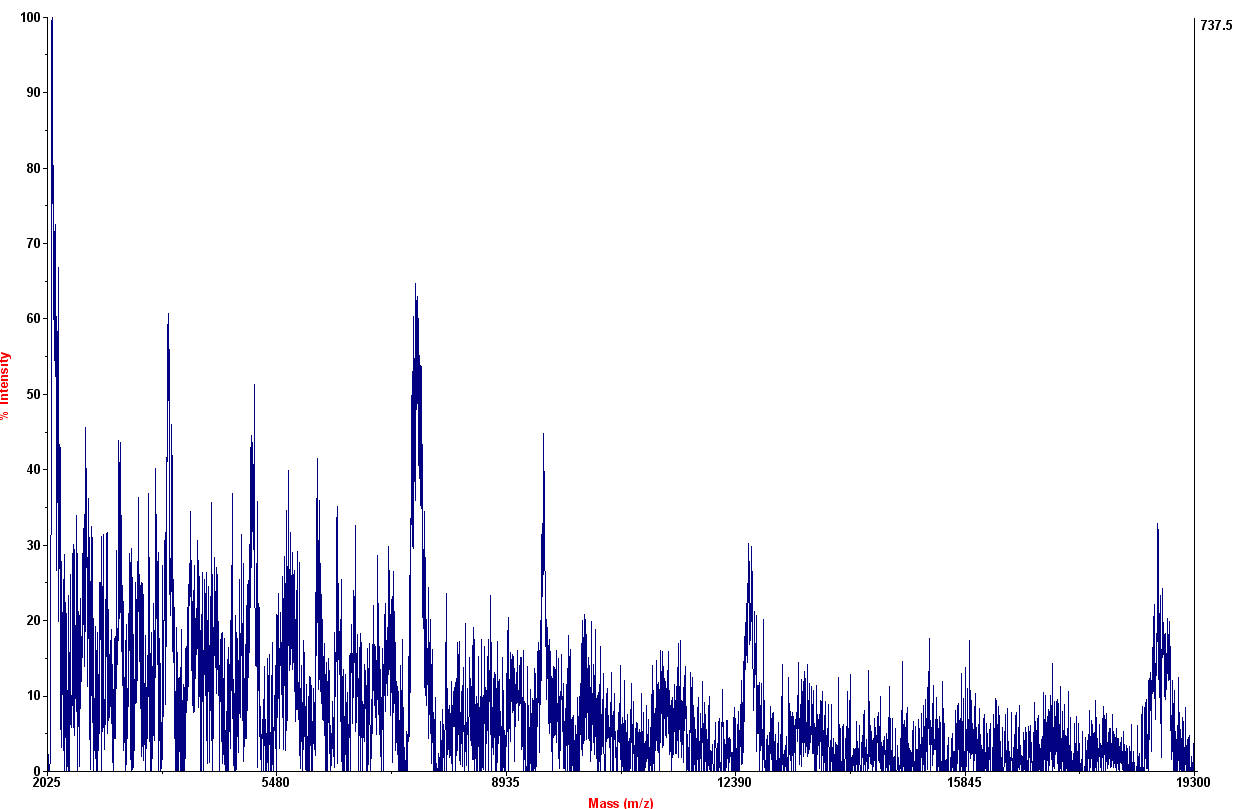

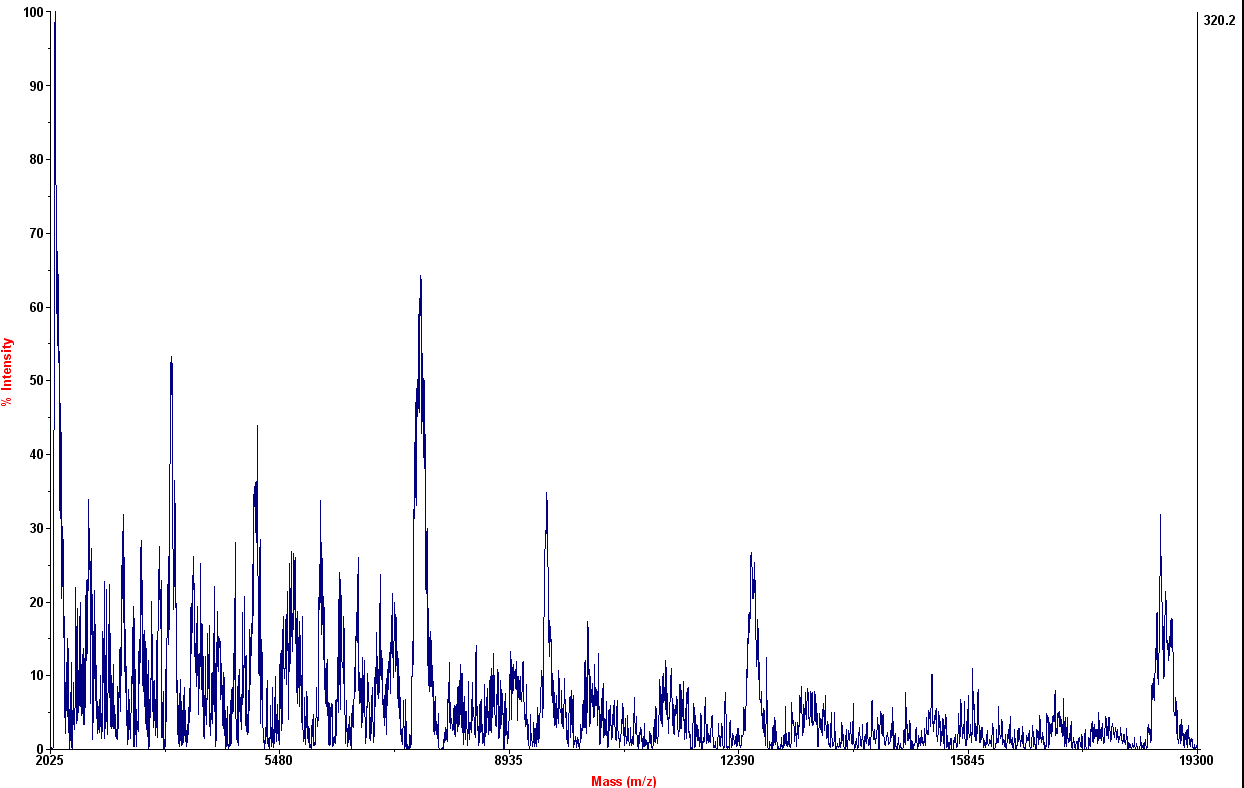


**Figure S3**: Mass spectrum of 3 repetitions of *Brucella melitensis* 50 CFU/mL dilution after interaction with the substrate


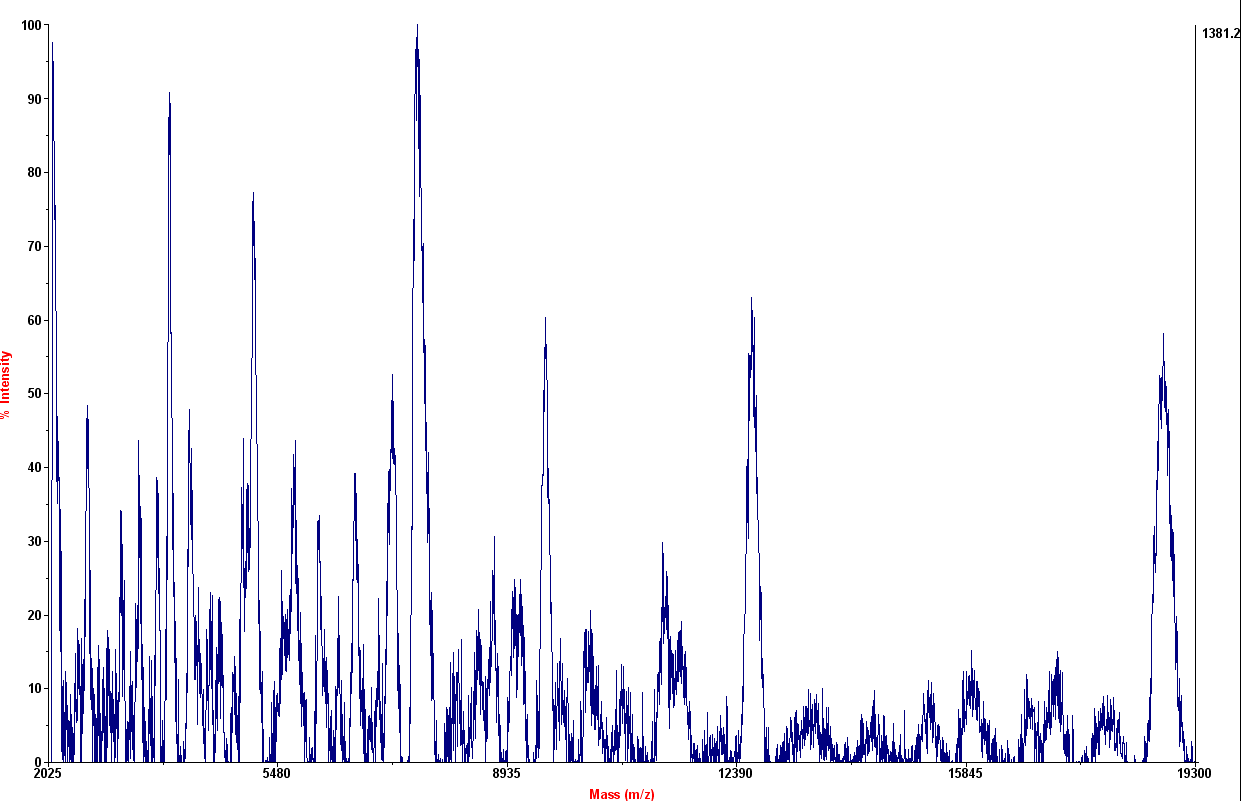

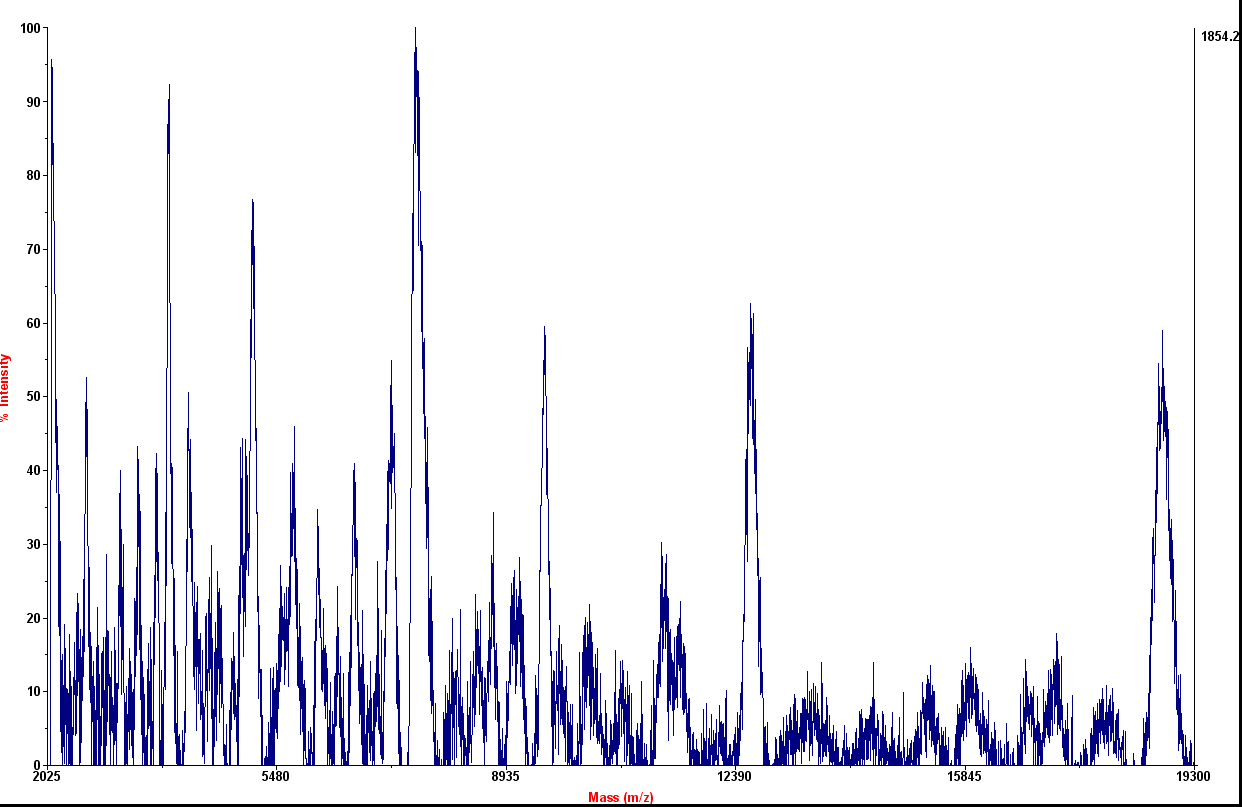

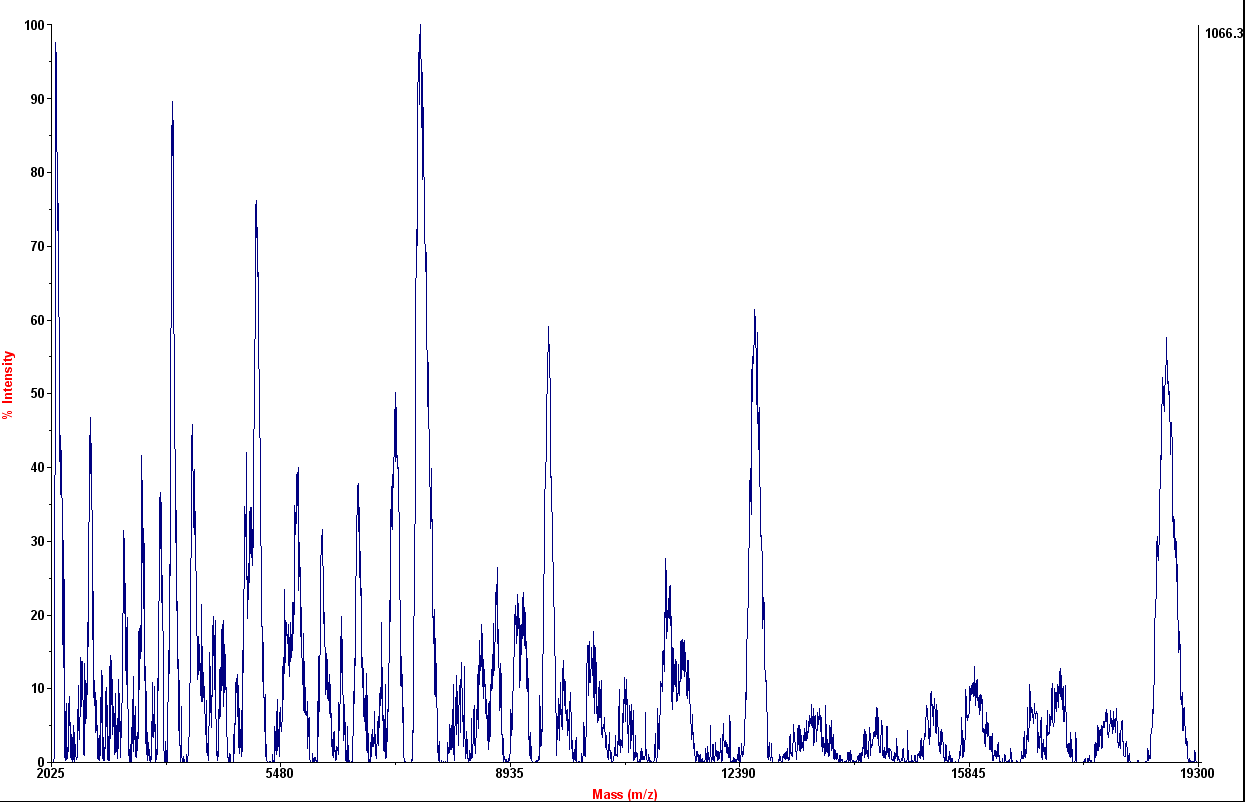


**Figure S4**: Mass spectrum of 3 repetitions of *Brucella melitensis* 500 CFU/mL dilution after interaction with the substrate


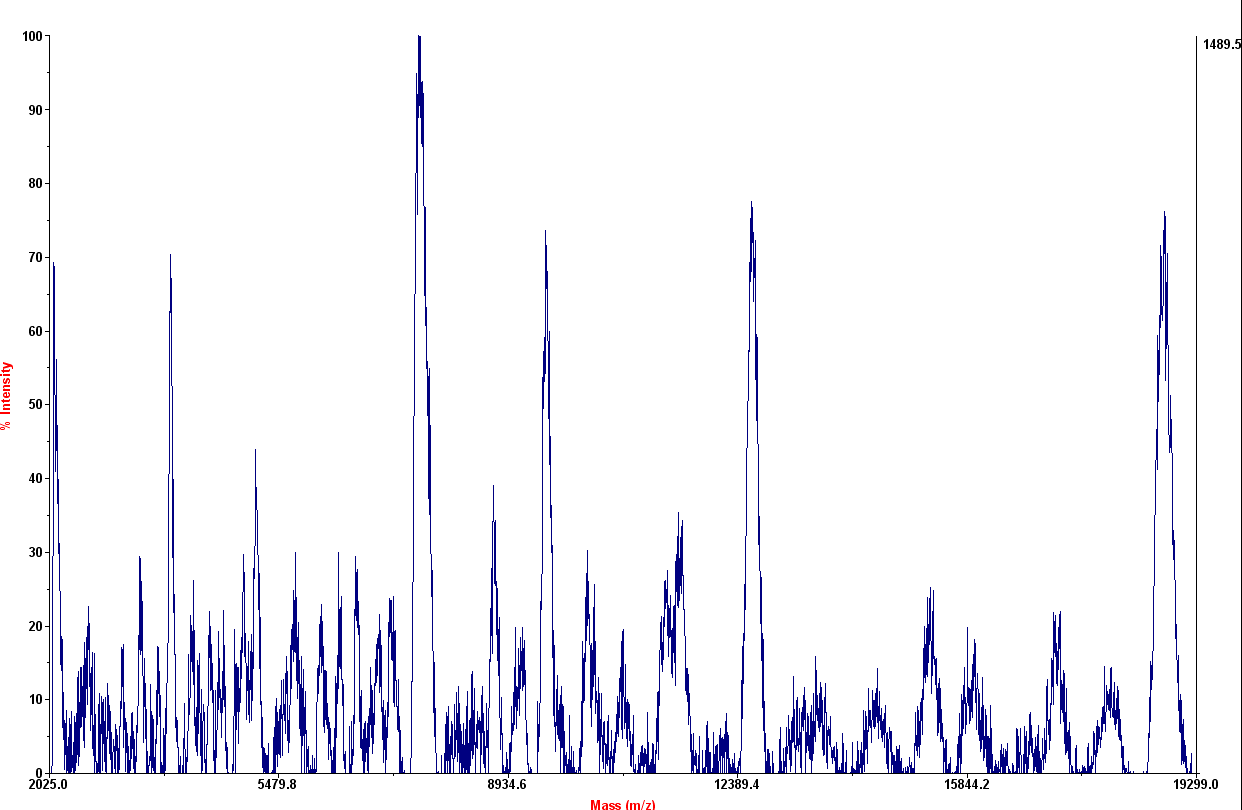

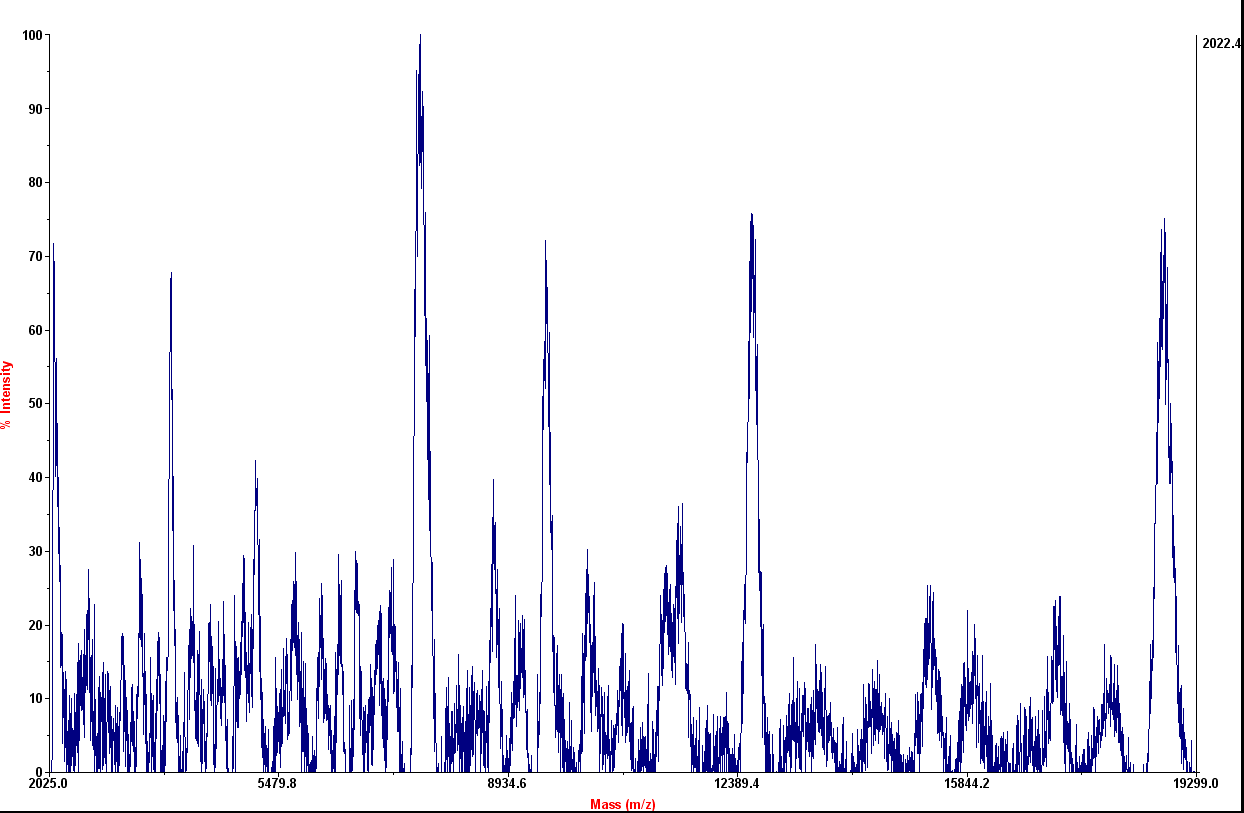

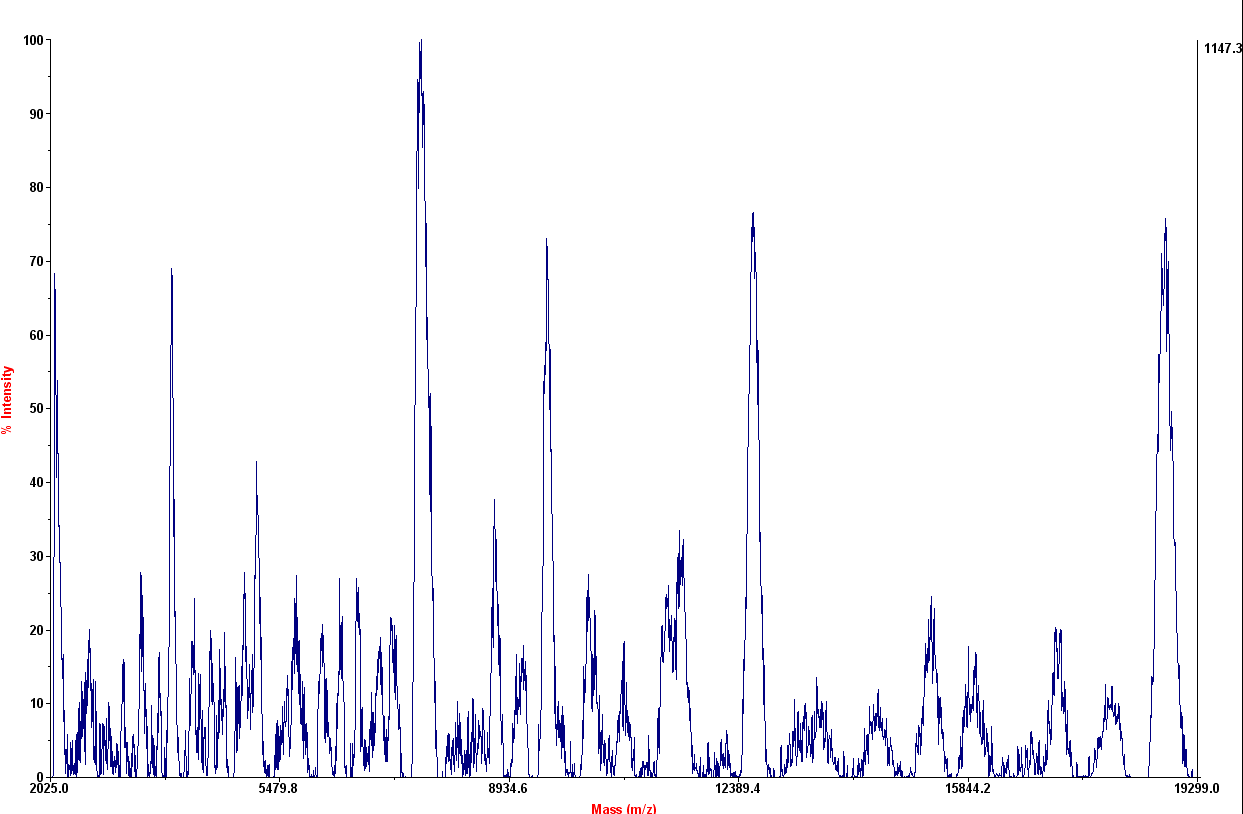


**Figure S5**: Mass spectrum of 3 repetitions of *Brucella melitensis* 5000 CFU/mL dilution after interaction with the substrate


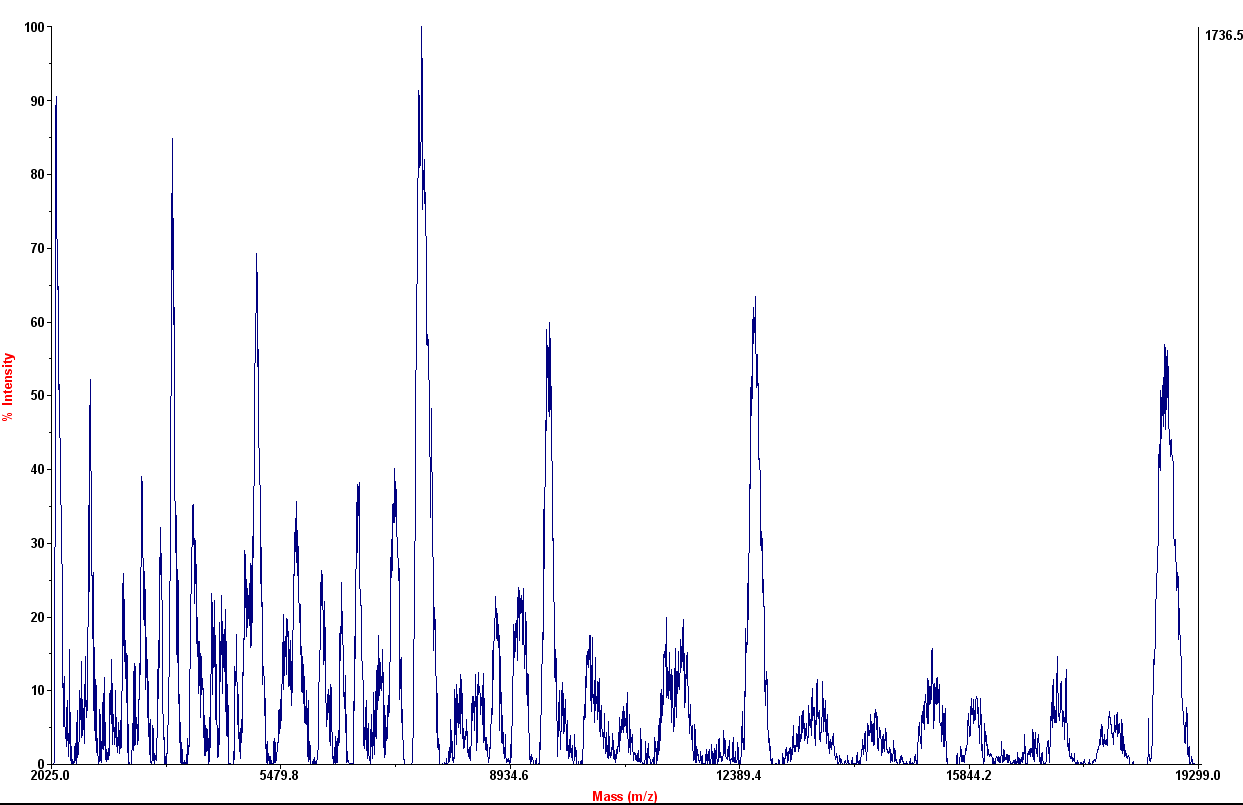

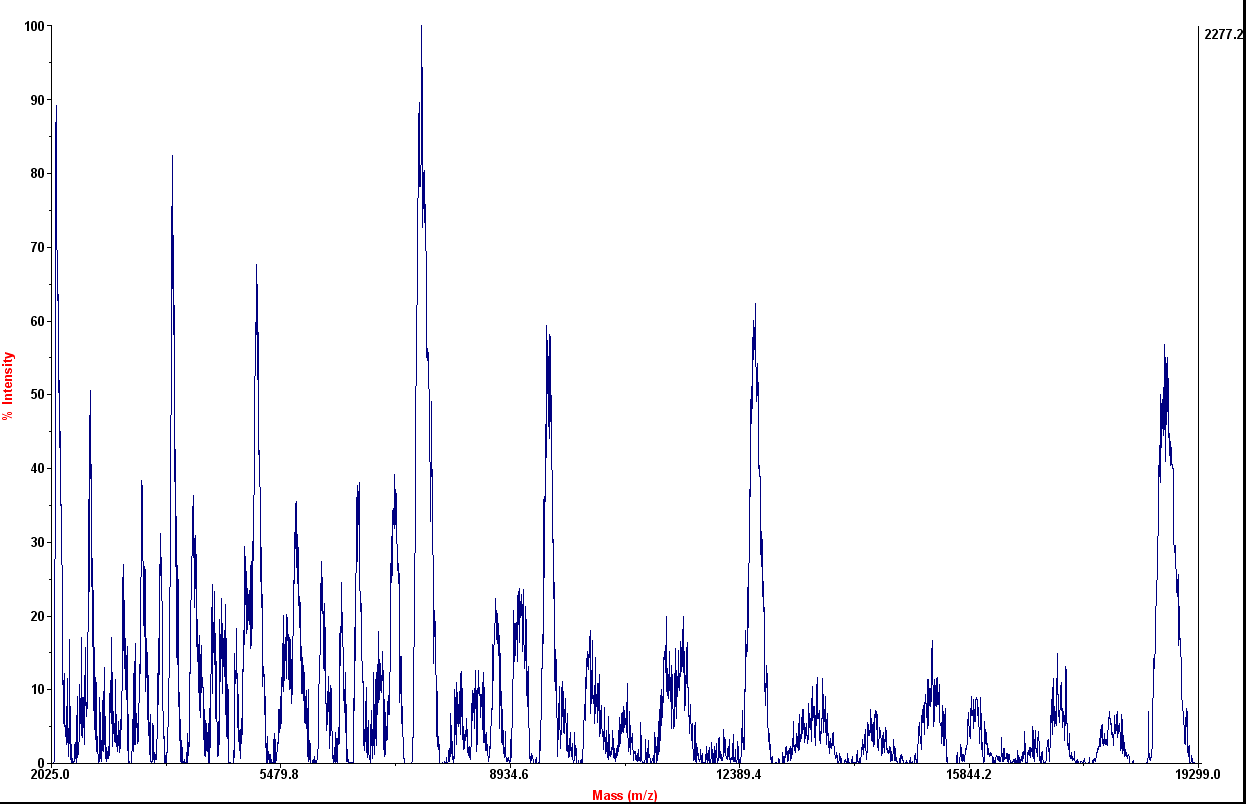

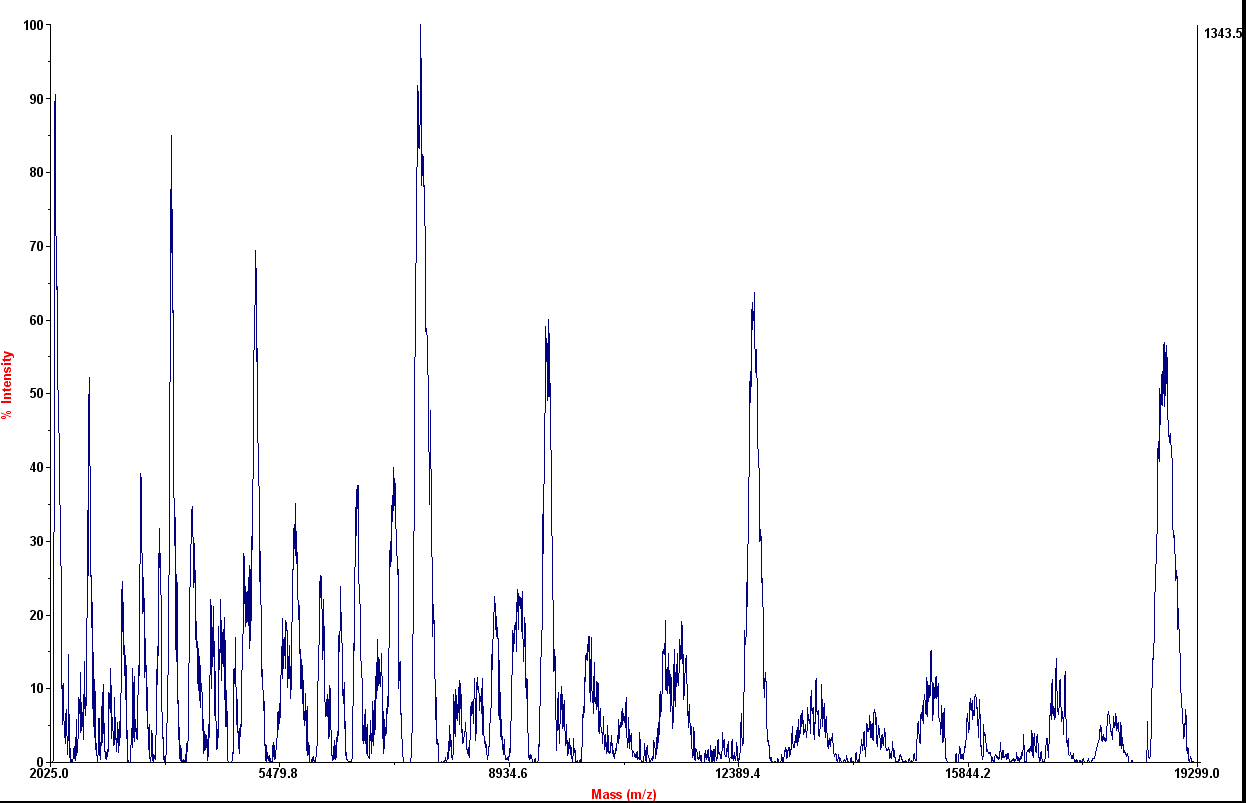


**Figure S6**: Mass spectrum of 3 repetitions of *Brucella melitensis* 50000 CFU/mL dilution after interaction with the substrate


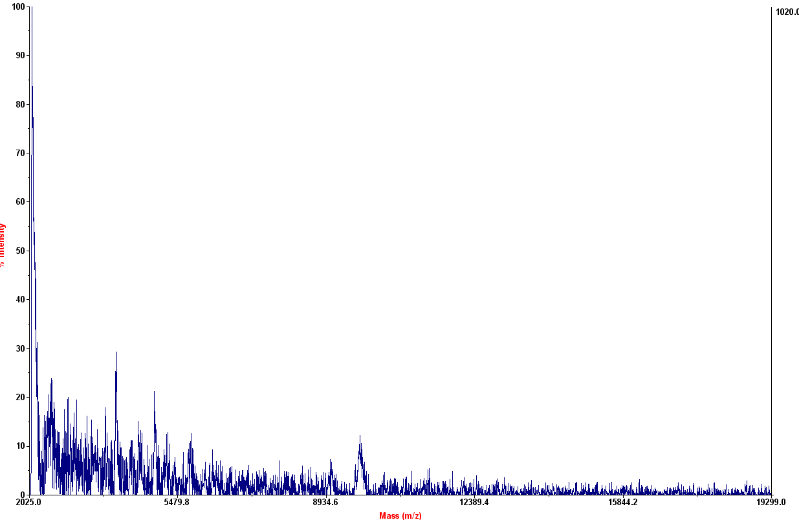

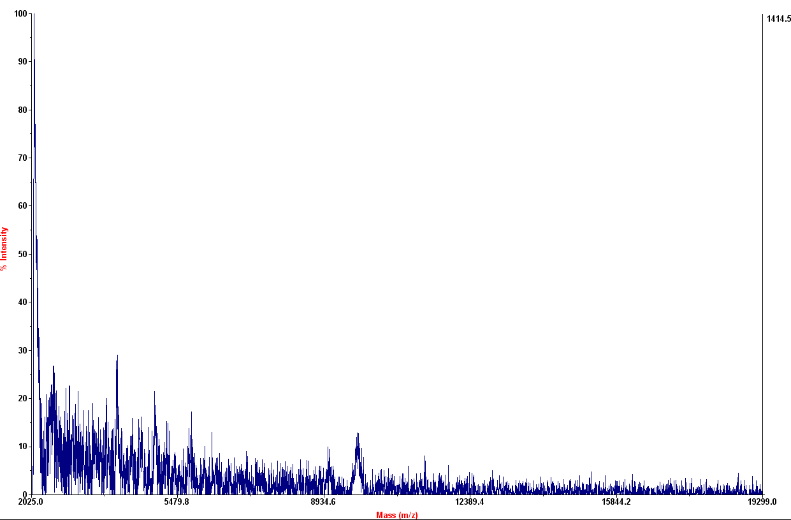

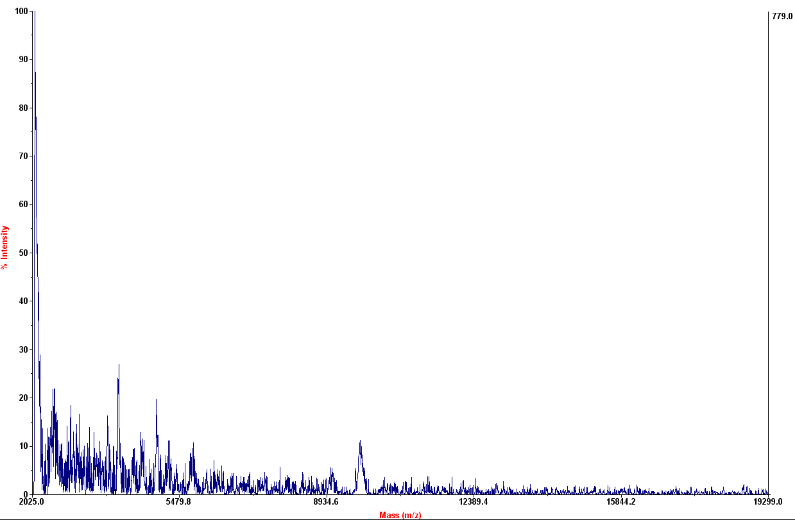


**Figure S7**: Mass spectrum of 3 repetitions of *Brucella melitensis* 50000 CFU/mL dilution without interaction.


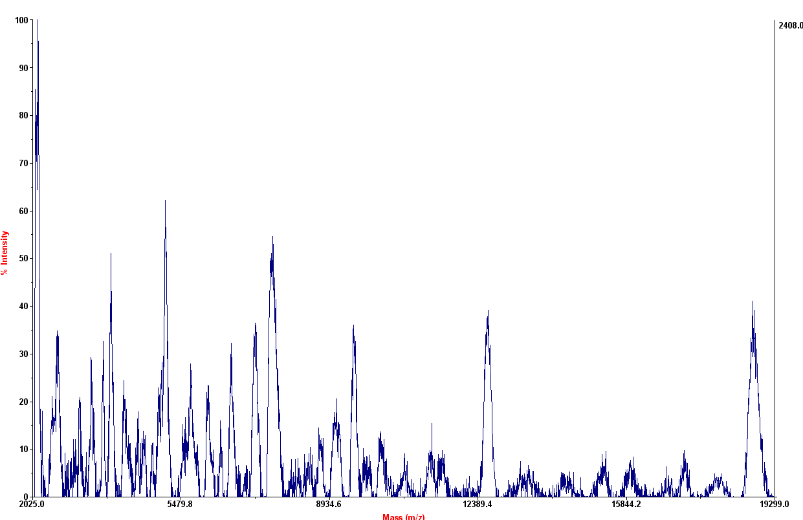

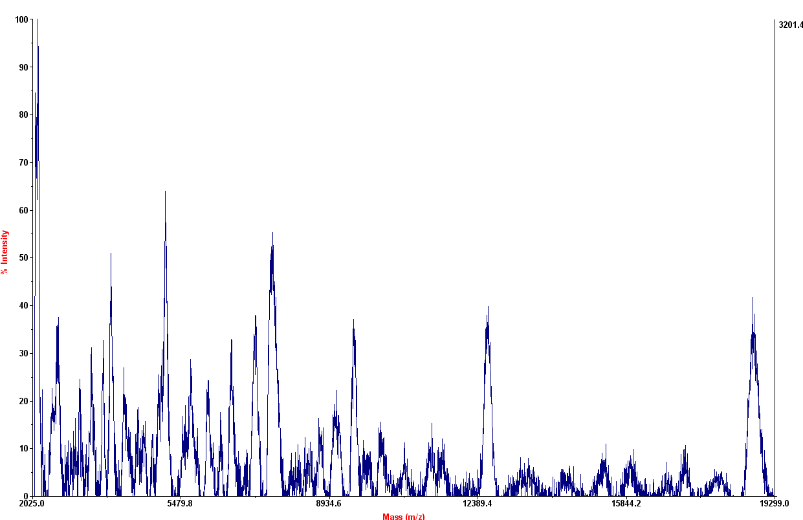

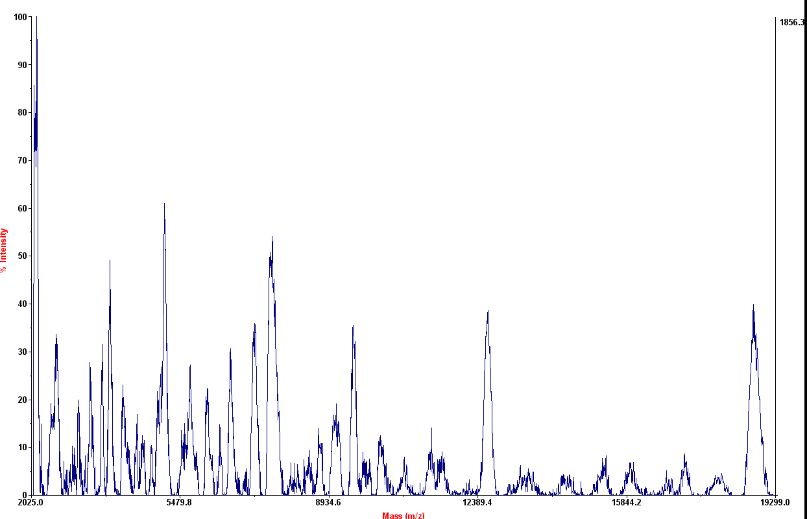


**Figure S8**: Mass spectrum of 3 repetitions of *Brucella melitensis* 500000 CFU/mL dilution after interaction with the substrate.


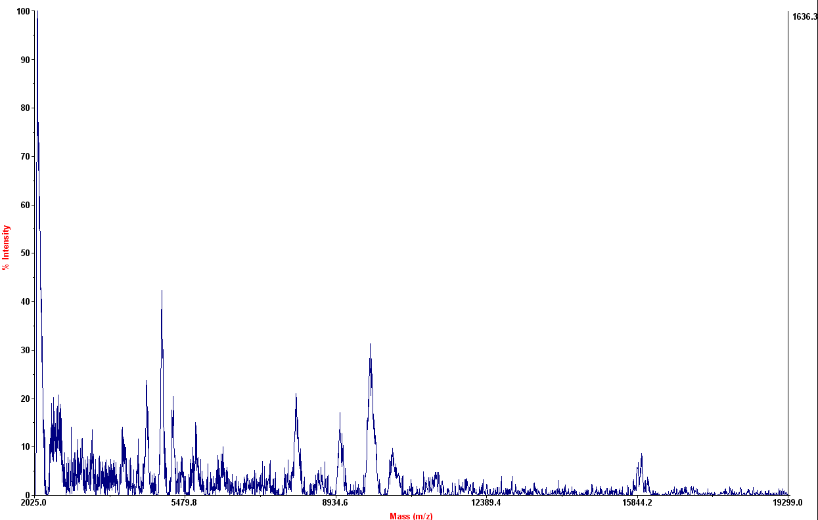

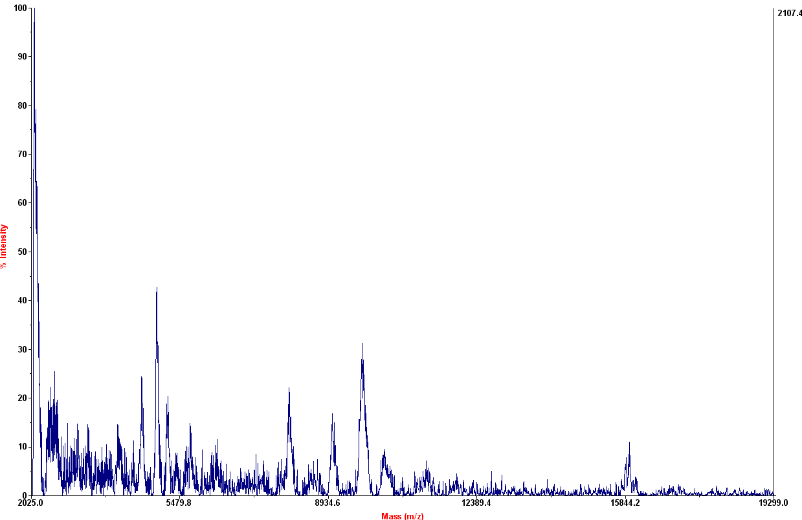

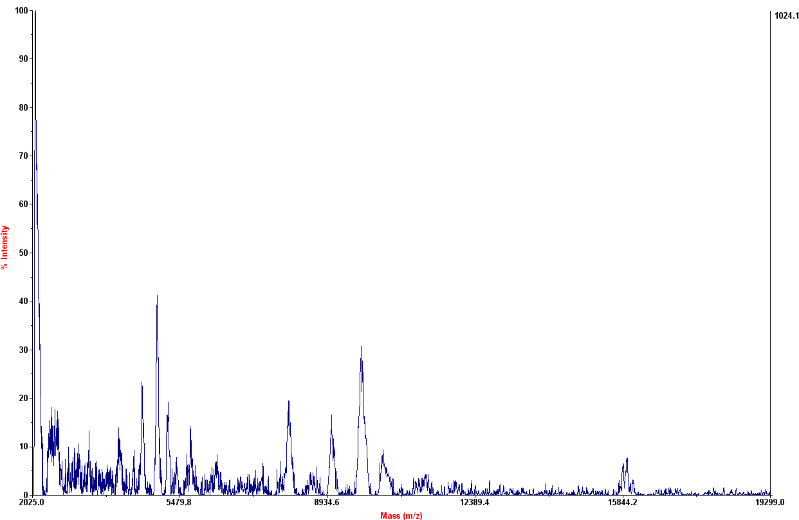


**Figure S9**: Mass spectrum of 3 repetitions of *Brucella melitensis* 500000 CFU/mL dilution without interaction.


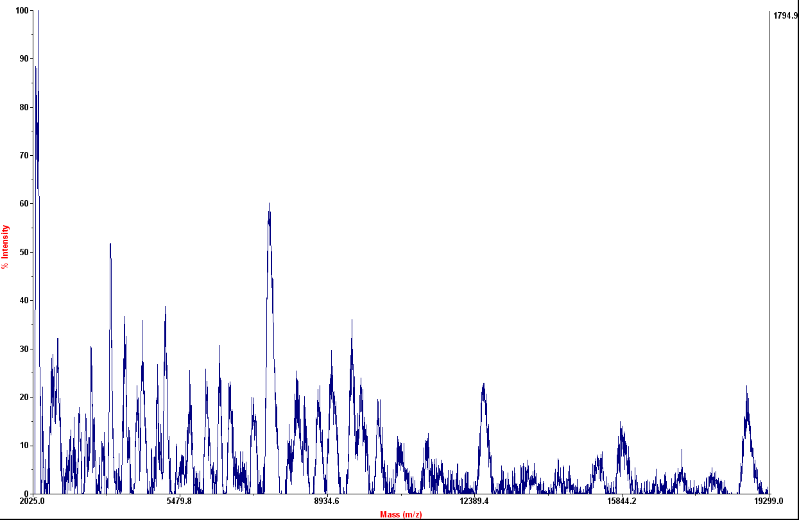

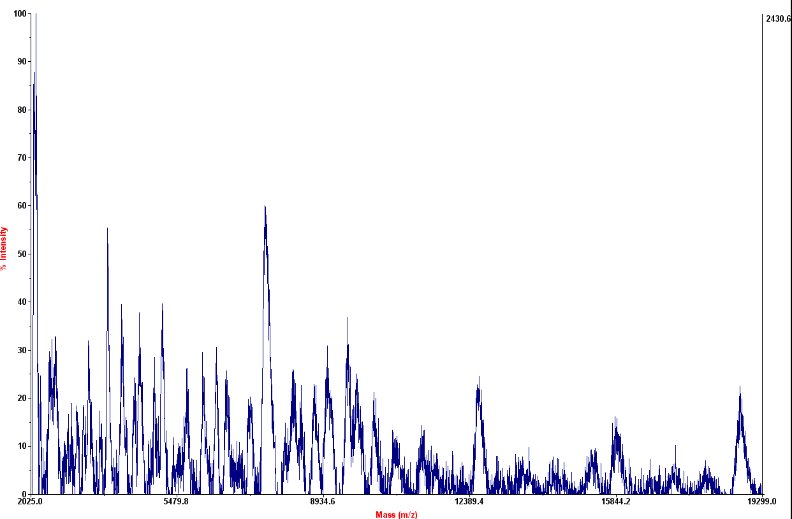

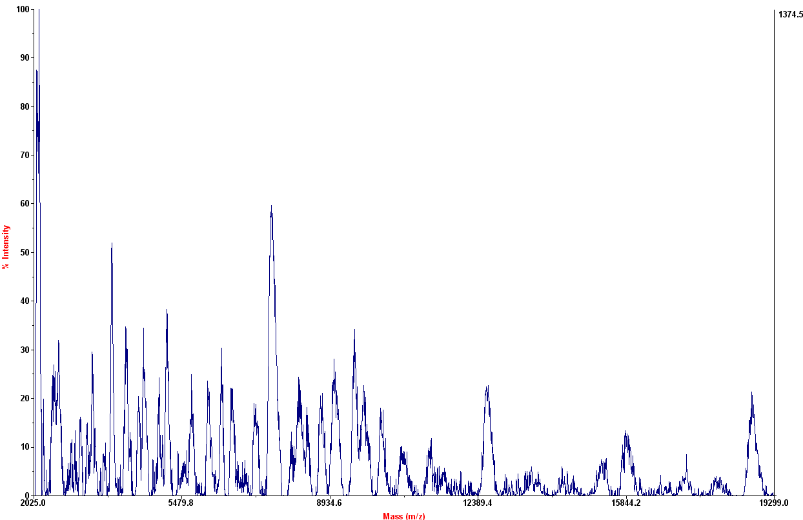


**Figure S10**: Mass spectrum of 3 repetitions of *Brucella melitensis* 5000000 CFU/mL dilution after interaction with the substrate.


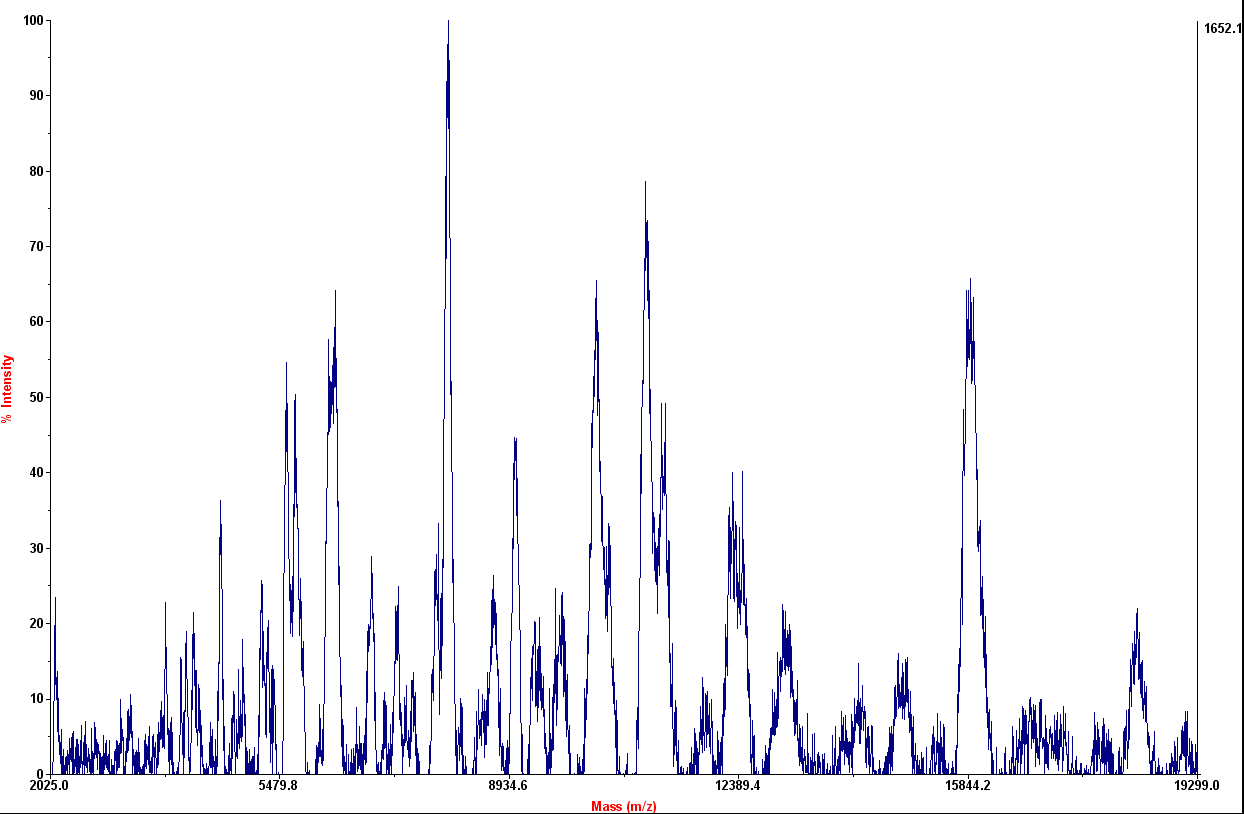

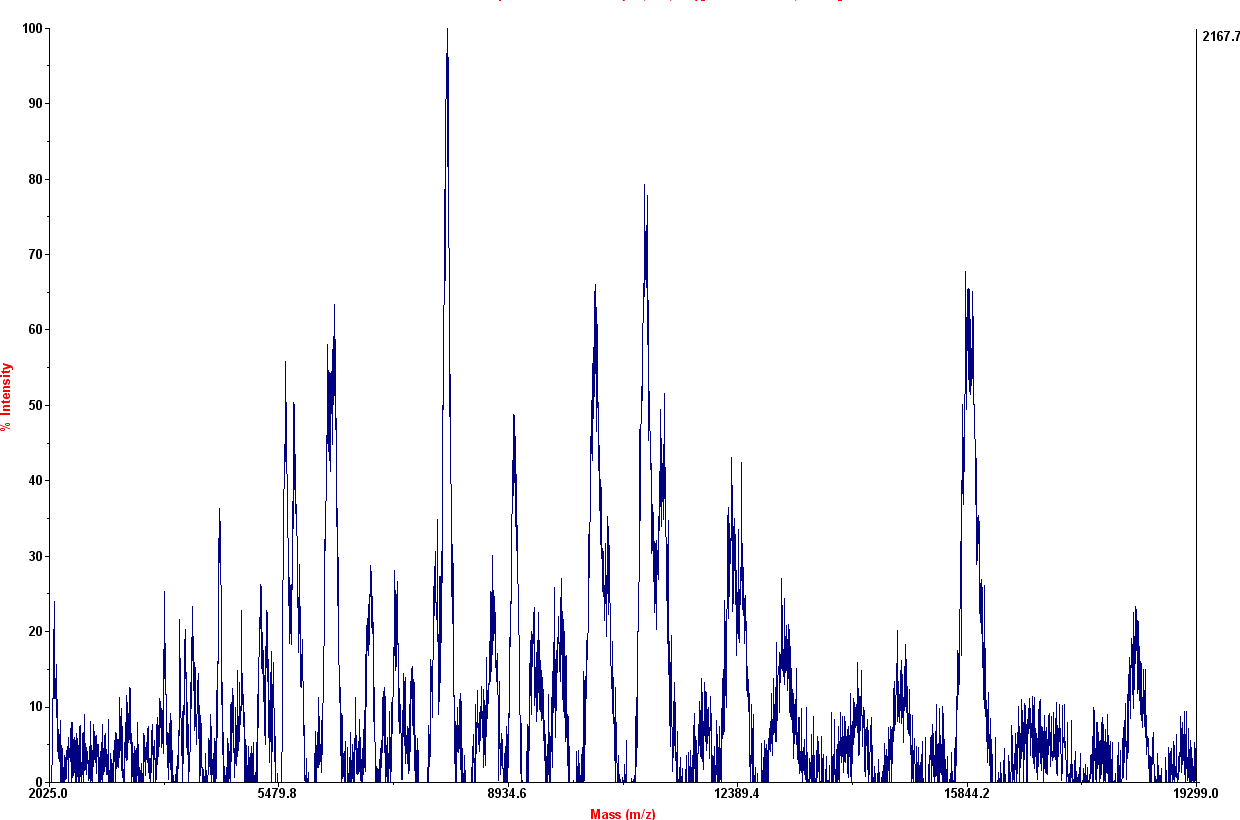

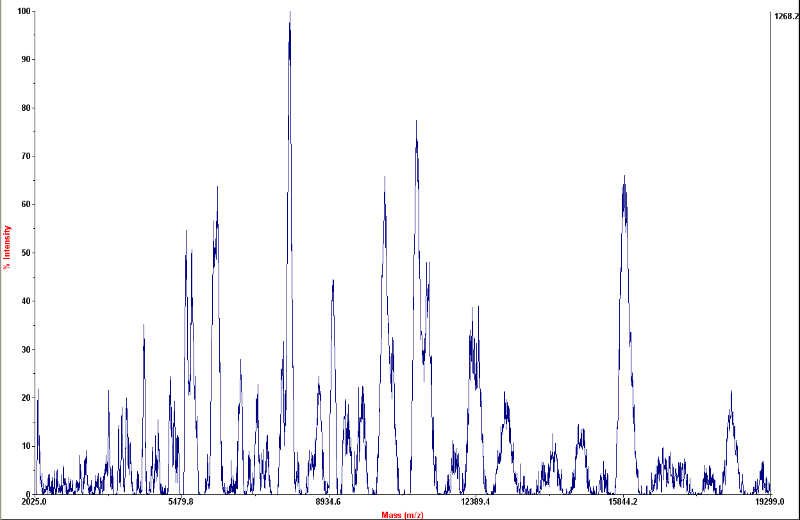


**Figure S11**: Mass spectrum of 3 repetitions of *Brucella melitensis* 5000000 CFU/mL dilution without interaction.


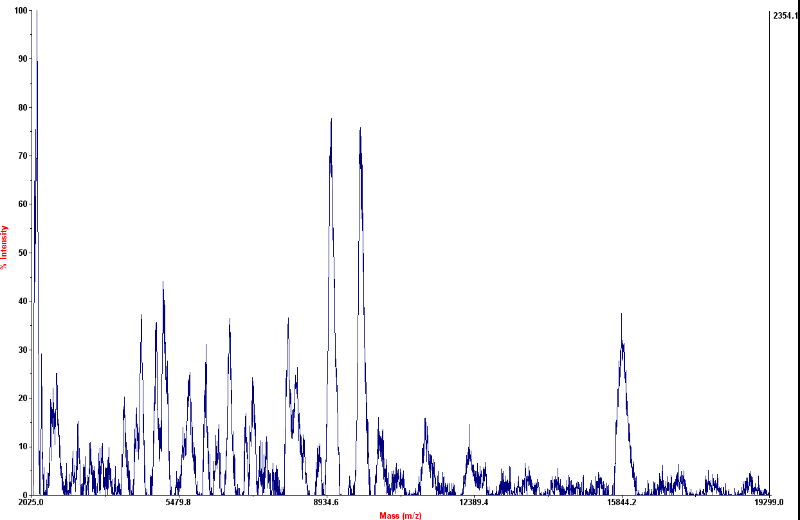

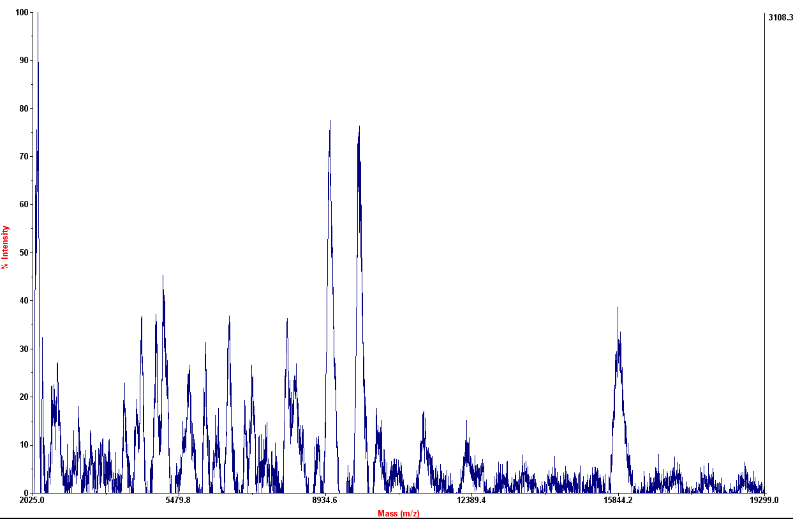

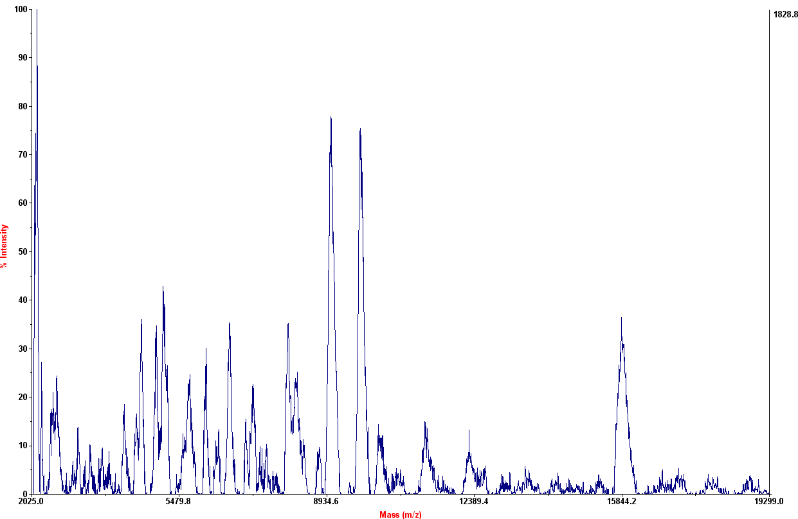


**Figure S12**: Mass spectrum of 3 repetitions of *Brucella melitensis* 50000000 CFU/mL dilution after interaction with the substrate.


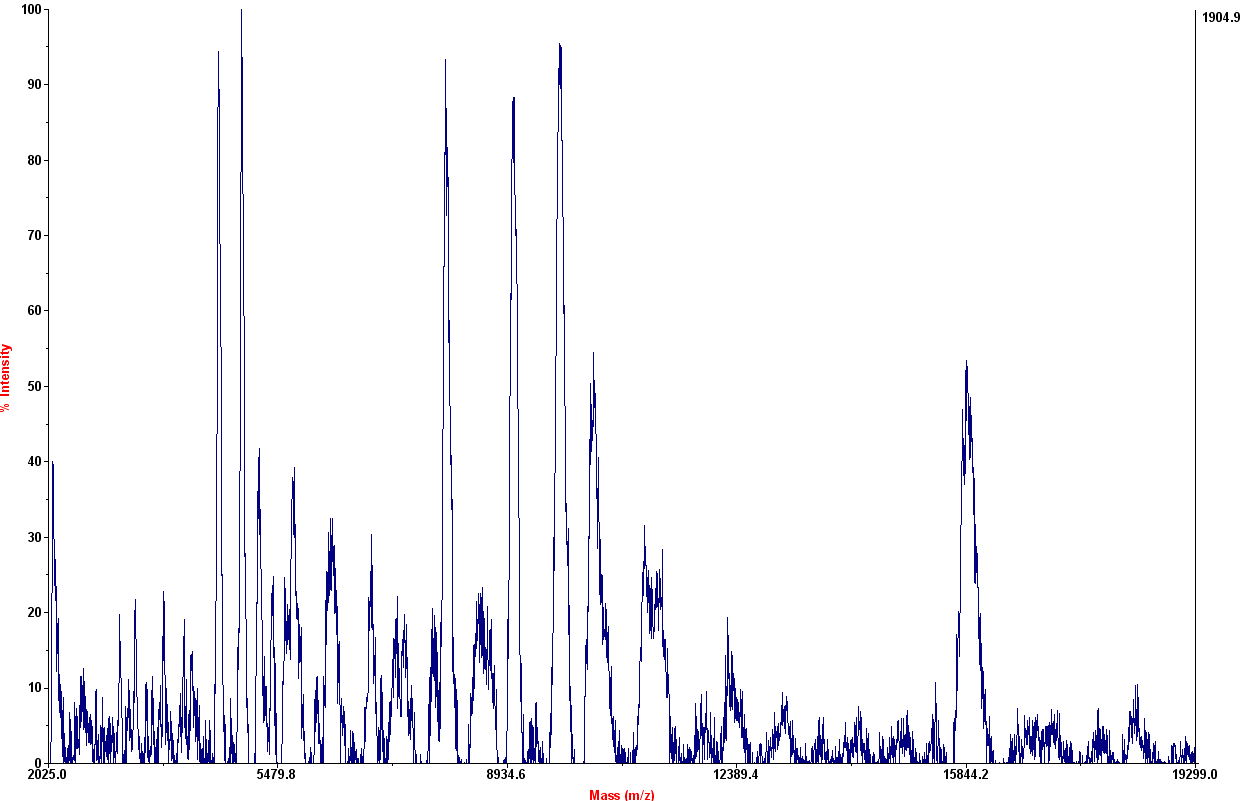

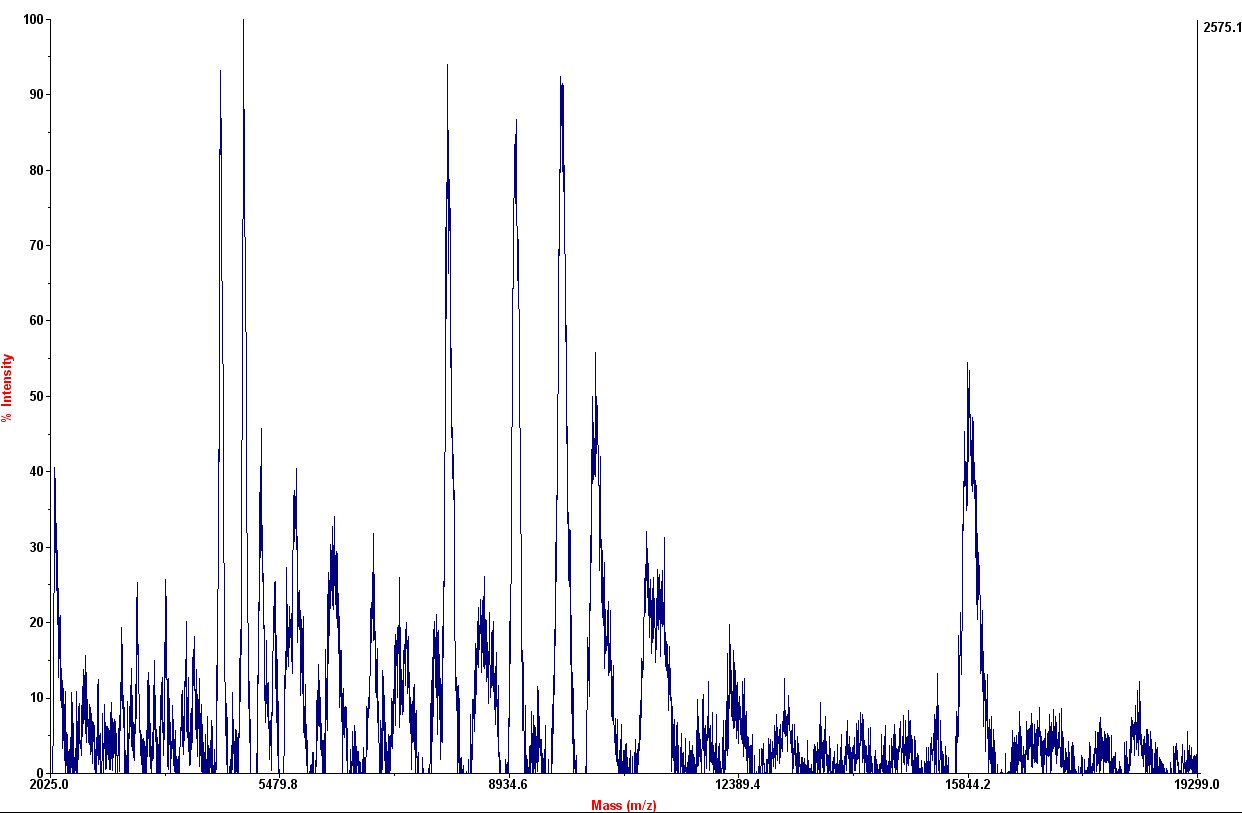

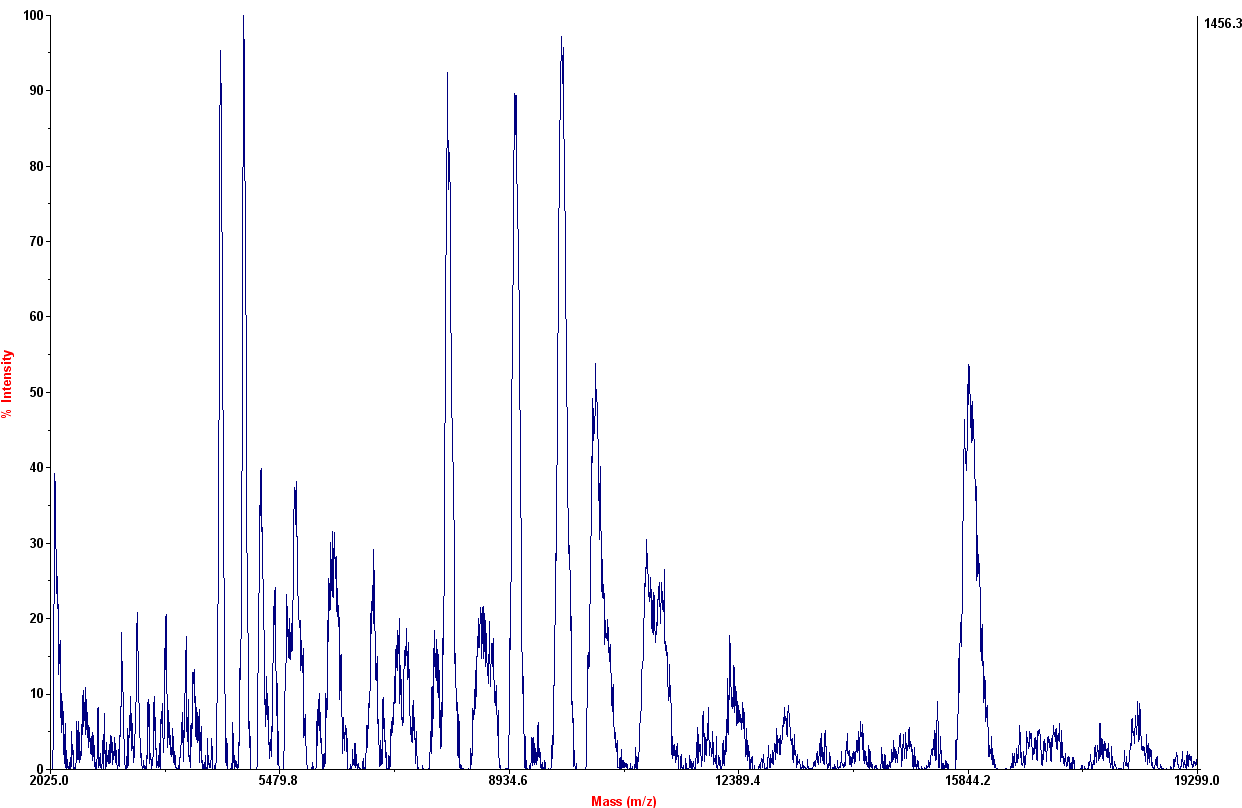


**Figure S13**: Mass spectrum of 3 repetitions of *Brucella melitensis* 50000000 CFU/mL dilution without interaction.


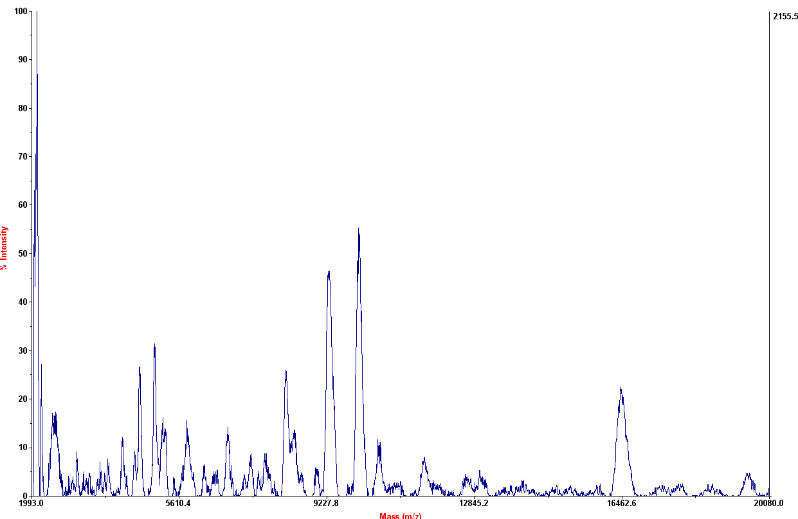

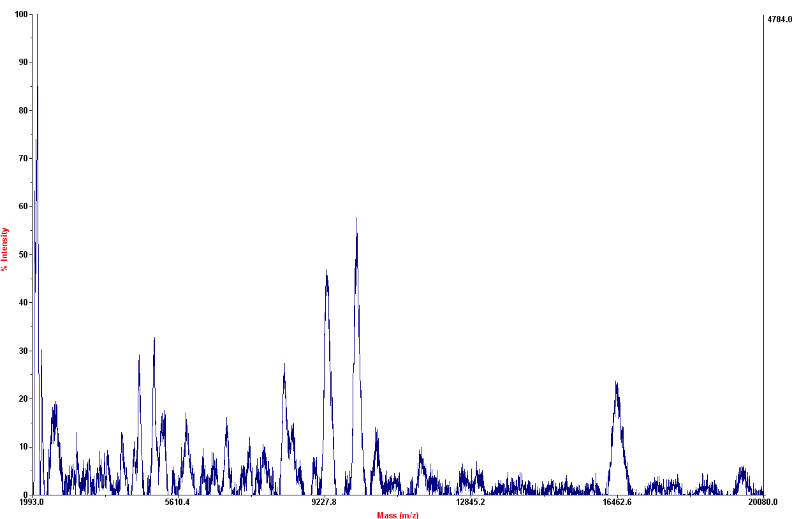

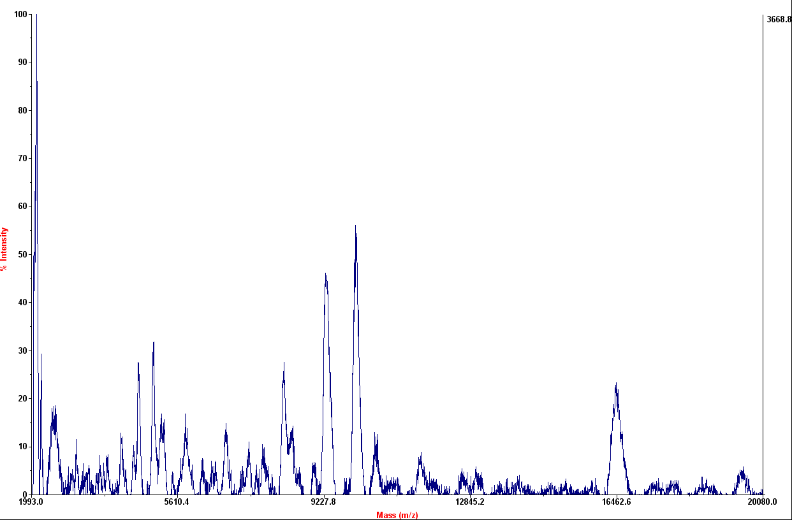


**Figure S14**: Mass spectrum of 3 repetitions of *Brucella melitensis* 500000000 CFU/mL dilution after interaction with the substrate.


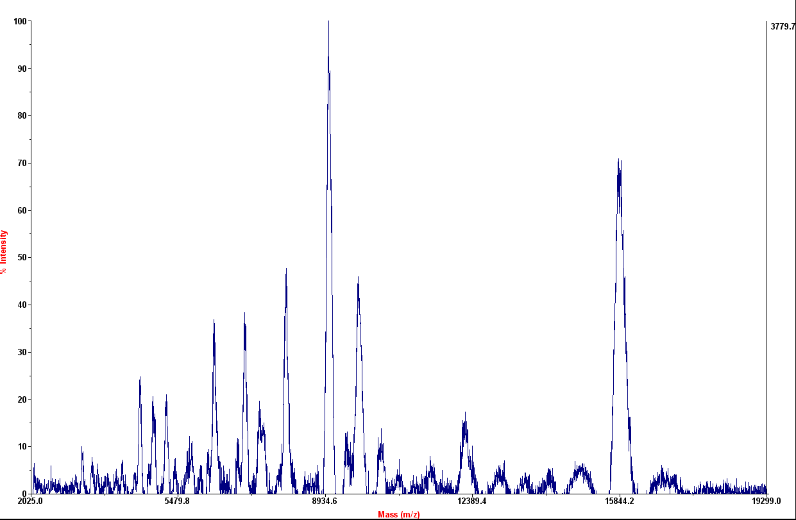

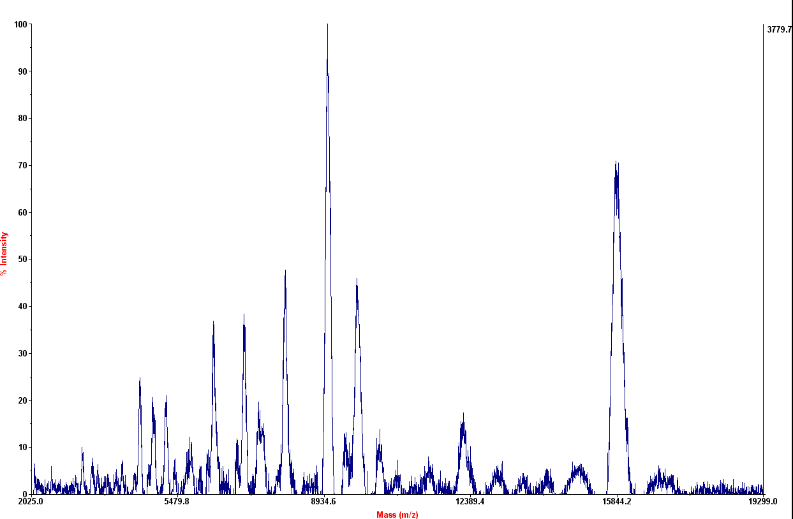

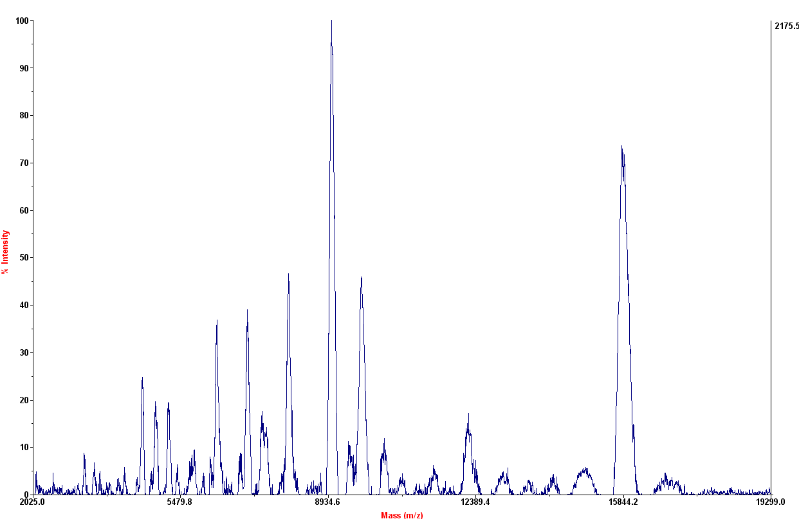


**Figure S15**: Mass spectrum of 3 repetitions of *Brucella melitensis* 500000000 CFU/mL dilution without interaction.
